# Novel genetic insight for psoriasis: integrative genome-wide analyses in 863 080 individuals and proteome-wide Mendelian randomization

**DOI:** 10.1093/bib/bbaf032

**Published:** 2025-01-30

**Authors:** Shunying Liu, Lingfei Li, Yi Liang, Yang Tan, Xiaoyu Wang, Yanhai Feng, Nian Chen, Xia Lei

**Affiliations:** Department of Dermatology, Daping Hospital, Army Medical University, No. 10, Changjiang Branch Road, Yuzhong District, Chongqing 400042, China; Research Center for Skin Tissue Engineering of Chongqing Higher Education Institutions, Daping Hospital, Army Medical University, No. 10, Changjiang Branch Road, Yuzhong District, Chongqing 400042, China; Department of Dermatology, Daping Hospital, Army Medical University, No. 10, Changjiang Branch Road, Yuzhong District, Chongqing 400042, China; Research Center for Skin Tissue Engineering of Chongqing Higher Education Institutions, Daping Hospital, Army Medical University, No. 10, Changjiang Branch Road, Yuzhong District, Chongqing 400042, China; Department of Dermatology, Daping Hospital, Army Medical University, No. 10, Changjiang Branch Road, Yuzhong District, Chongqing 400042, China; Research Center for Skin Tissue Engineering of Chongqing Higher Education Institutions, Daping Hospital, Army Medical University, No. 10, Changjiang Branch Road, Yuzhong District, Chongqing 400042, China; Department of Dermatology, Daping Hospital, Army Medical University, No. 10, Changjiang Branch Road, Yuzhong District, Chongqing 400042, China; Research Center for Skin Tissue Engineering of Chongqing Higher Education Institutions, Daping Hospital, Army Medical University, No. 10, Changjiang Branch Road, Yuzhong District, Chongqing 400042, China; Department of Dermatology, Daping Hospital, Army Medical University, No. 10, Changjiang Branch Road, Yuzhong District, Chongqing 400042, China; Research Center for Skin Tissue Engineering of Chongqing Higher Education Institutions, Daping Hospital, Army Medical University, No. 10, Changjiang Branch Road, Yuzhong District, Chongqing 400042, China; Department of Dermatology, Daping Hospital, Army Medical University, No. 10, Changjiang Branch Road, Yuzhong District, Chongqing 400042, China; Research Center for Skin Tissue Engineering of Chongqing Higher Education Institutions, Daping Hospital, Army Medical University, No. 10, Changjiang Branch Road, Yuzhong District, Chongqing 400042, China; Army 953 Hospital, Shigatse Branch of Xinqiao Hospital, Army Medical University, No. 5, Mount Everest West Road, Xigaze District, Shigatse 857007, China; Department of Dermatology, Daping Hospital, Army Medical University, No. 10, Changjiang Branch Road, Yuzhong District, Chongqing 400042, China; Research Center for Skin Tissue Engineering of Chongqing Higher Education Institutions, Daping Hospital, Army Medical University, No. 10, Changjiang Branch Road, Yuzhong District, Chongqing 400042, China; Department of Dermatology, Daping Hospital, Army Medical University, No. 10, Changjiang Branch Road, Yuzhong District, Chongqing 400042, China; Research Center for Skin Tissue Engineering of Chongqing Higher Education Institutions, Daping Hospital, Army Medical University, No. 10, Changjiang Branch Road, Yuzhong District, Chongqing 400042, China

**Keywords:** psoriasis, GWAS-meta, Mendelian randomization, proteome, genetics

## Abstract

Psoriasis affects a significant proportion of the worldwide population and causes an extremely heavy psychological and physical burden. The existing therapeutic schemes have many deficiencies such as limited efficacies and various side effects. Therefore, novel ways of treating psoriasis are urgently needed. A large-scale meta-analysis of psoriasis genome-wide association studies (GWAS) totaling 20 105 cases and 842 975 controls was conducted. Based on the GWAS results, Mendelian randomization (MR) analyses were then performed on three *cis*-protein quantitative trait loci (pQTL) data in blood. Furthermore, druggability verification and mouse knock-out models were utilized to explore the clinical value of screened proteins. We identified 42 genome-wide significant psoriasis risk variants (*P* < 5 × 10^−8^), of which 33 were previously unreported. MR analyses unveiled 19 unique circulating proteins that were associated with psoriasis, among which only AIF1, FCGR3A, NEU1, HSPA1A, TNXB, and ABO were the potential proteins that interacted with psoriasis risk after being analyzed with high evidence of colocalization (PP.H4 > 0.9). In addition, AIF1, FCGR3A, and HSPA1A have been finally determined to be feasible therapeutic targets for psoriasis after being confirmed by druggability verification and specific mouse knock-out models. This large-scale GWAS meta-analysis identified 33 new variants for psoriasis. This study announced that AIF1, FCGR3, and HSPA1A were the unexplored but material variants of psoriasis, thus providing novel and valuable targets for psoriasis treatment and broadening new orientation of drug development for psoriasis.

## Introduction

Psoriasis is a chronic, immune-mediated, noninfectious disease, affecting people of all ages [1]. In recent years, ~125 million people worldwide are diagnosed, with prevalence in the adult population ranging from 30.3 per 100 000 in Taiwan to 321 per 100 000 in Italy [2, 3]. It can affect all parts of the skin and systems throughout the body, potentially resulting in a poor prognosis, psychological burden, and social stigma [4]. The main therapeutic schemes for psoriasis are topical therapy for mild psoriasis and systemic drug therapy based on biological agents for moderate or severe psoriasis [5]. However, in clinical practice, some patients do not acquire the expected response to the drug, which may be caused by the ineffective drug [6]. Therefore, more targeted and effective drugs need to be developed.

Early research has shown that gene-validated drug targets are more likely to be successfully developed as clinical drugs [7]. Genome-wide association studies (GWASs) identify variants that are strictly associated with clinically relevant phenotypes and reveal susceptibility genes, mechanisms in psoriasis, and the genetic overlap exhibited between different traits [8]. GWASs have recently identified risk loci for psoriasis [9–14]. For example, the IL12B variant and IL23R variant were found to be associated with psoriasis susceptibility [15], which revealed important biological and genetic insights for suitable drug–target compounds. However, the total sample is relatively small, and the significant single-nucleotide polymorphisms (SNPs) for psoriasis are limited.

Circulating proteins, including cytokines, growth factors, and proteases, play pivotal roles in psoriasis pathophysiology [16]. For example, by plasma metabolomic analysis, Qian *et al*. recognized estrogen receptor 1 (ESR1), opioid receptor mu 1 (OPRM1), and hydroxysteroid 11-beta dehydrogenase 1 (HSD11B1) as promising therapeutic targets for psoriasis [17]. To achieve this purpose, Mendelian randomization (MR) is frequently employed. MR can combine SNP–protein expression with SNP–disease association to estimate the causal effects of exposures on disease outcomes. Excluding confounding bias or reverse causation, large-scale protein quantitative trait locus (pQTL) data in MR studies can provide a deeper understanding of genetic pathogenetic factors associated with complex diseases [18]. At present, studies using MR to identify therapeutic targets for diseases have been reported, such as stroke, multiple sclerosis, and heart failure [19–21]. However, MR has not yet been used to find therapeutic targets for psoriasis.

In the current study, based on large-scale plasma proteome pQTL and GWAS summary statics, we performed : (i) a GWAS meta-analysis using the three largest GWAS datasets for a total of 20 105 cases and 842 975 controls; (ii) a proteome-wide MR studies based on three *cis*-pQTLs; (iii) a colocalization analysis for further associated protein identification; and (iv) an estimation of potential drug targetability of these associated proteins. AIF1 (allograft inflammatory factor 1), FCGR3, and HSPA1A [shock protein family A (Hsp70) member 1A] were finally identified as novel and valuable targets for psoriasis treatment, broadening new possible orientations of drug development for psoriasis.

## Materials and methods

### Ethics and research design

**Figure 1:**
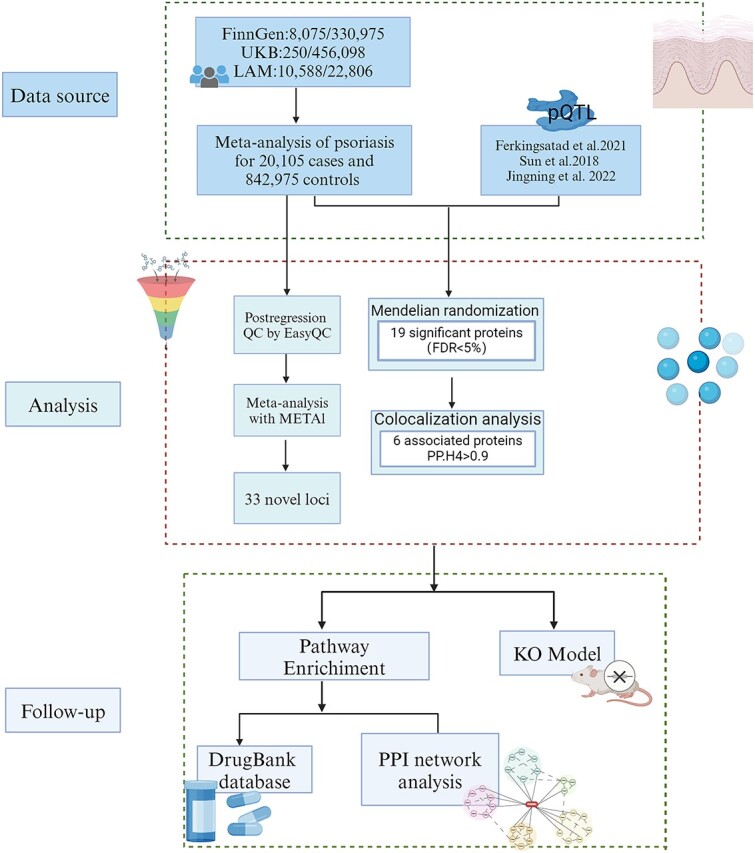
Study design and workflow. FDR, false discovery rate; KO model, knock-out model; PPI, protein–protein interaction; pQTL, protein quantitative trait loci. Scheme was created with Biorender.com.

The study is a secondary analysis from publicly available data. Ethical approval and informed consent were obtained from the participants and investigators of the original study. A flow diagram is shown in Fig. 1.

### Exposure data source

Three large-scale pQTLs were contained in our study. The pQTLs included the following: first, 4719 aptamers proteins in 35 559 Icelanders were measured by Ferkingstad *et al*. [22]. The second pQTL data with levels of 4907 circulating proteins were extracted from 7213 European Americans [23]. The last pQTL data were obtained from the INTERVAL study and measured 3622 plasma proteins from 3301 European populations [24]. Details of the three studies are presented in Supplementary Table S1.

We applied the following strategies to filter the cis-pQTLs and identify potential candidate genes: (i) SNPs associated with any protein were selected (*P* < 5 × 10^−8^); (ii) the linkage disequilibrium (LD) clumping was then conducted to identify independent pQTLs for each protein (*r*^2^ < 0.001); and (iii) we defined *cis*-pQTL when the leading SNP in the region, which was located within 500 kb of the transcription start site of the protein-coding gene.

### Outcome data sources

The genome-wide association study (GWAS) data for psoriasis included in the meta-analysis were from the FinnGen consortium, the GWAS catalog, and the UK Biobank (UKB), with a total of 828 792 participants (Supplementary Table S2). Detailedly, 8075 patients and 330 975 controls were extracted from the ninth release of the FinnGen consortium [12]. For the GWAS catalog data, GCST005527 was openly acquired, which consisted of 10 588 cases and 22 806 controls [13]. Besides, the remaining 456,348 individuals were screened from the large-scale UKB using the fastGWA-GLMM method [14]. These GWASs were all based on European populations and did not have known participant overlap with the pQTL studies.

### Genome-wide association meta-analysis for psoriasis

In this paper, EasyQC software [25] was used for quality control of GWAS data from the above three databases. Compared to the 1000 Genomes reference panel, variants with minor allele frequency < 1% or allele frequency difference > 0.2 were removed. All the data that passed the quality control were meta-analyzed by METAL software [26], using the fixed effect model. LD score regression (LDSC) [27] was used for calculating regression intercept and heritability among psoriasis traits. To enhance the stability of our results, we conducted a subsequent meta-analysis incorporating data from the UKB and FinnGen and validated our findings using an independent cohort. We have also considered the complexities introduced by LD proxies, specifically focusing on variants with an *R*2 > 0.7 within a 500 000-bp window [28, 29].

### Proteome-wide Mendelian randomization

MR analysis was performed by using the R package “TwoSampleMR” [30]. The research screened available pQTLs for MR analysis through genome-wide significance level (*P* < 5 × 10^−8^) and LD clustering (*r*^2^ < 0.001). In order to discover potential susceptibility proteins, the correlation between proteins and psoriasis was accounted for through various methods. For genes having one SNP, the Wald ratio method was used. For genes having over 1 SNP, the inverse variance weighting (IVW) model was employed. MR sensitivity analysis was also implemented to get more robust results. If the expose had over one available SNP, pleiotropy was assessed via the MR–Egger intercept and Cochran’s *Q*. False discovery rate (FDR)-corrected *P*-values were computed to establish significance thresholds of multiple testing. FDR < 0.05 was considered significant in the psoriasis-associated genes’ discovering phase and validation phase. We calculated the odds ratios (ORs), *P* value, and corresponding confidence intervals (CIs) using the Wald ratio (one SNP available) and the IVW method (more than one SNP available), respectively. Besides, the Steiger filtering test was conducted to ensure correctness of the causality between psoriasis and proteins.

### Colocalization analysis

Colocalization analysis was carried out using the “coloc” package [31] to verify whether a significant relationship between psoriasis and pQTLs is attributable to linkage imbalance. The analysis offers posterior probabilities for the following hypotheses: PP.H0 (all uncorrelated traits), PP.H1 (related gene expression and unrelated traits), PP.H2 (related traits and unrelated gene expression), PP.H3 (related traits and gene expression, with distinguishable causal variants), and PP.H4 (related traits and gene expression, with obscure causal variants). Proteins that passed the significant FDR threshold would be then subjected to colocalization analysis. Subsequently, PPH4 > 0.90 in this paper was set as the threshold value to achieve high-support significance [13]. LocusZoom was also applied to provide a fast visual display of colocalization results [32].

### Druggable proteome identification

To assess the druggability of identified proteins, abundant druggable targets in the DrugBank database were compared. These druggable proteins were considered therapeutic targets of the approved or clinical trial phase. Proteins that could not be found in the database were regarded as unapproved targets. This step also examined whether indications for approved targets were risk factors for psoriasis to avoid potential confounding bias.

Besides, the protein–protein interaction (PPI) network was used to explore the interactions between predicted psoriasis-associated proteins and well-established targets of medications. Proteins identified in previous work were roundly acquired through OpenTargets [33]. PPI networks complied using the STRING database (version 11.5), with the required interaction score >0.4.

### Mouse knock-out models for potential proteins through the Mouse Genome Informatics database

Integrating information from open genomic resources and published experimental data, the Mouse Genome Informatics (MGI) resource constantly provides data on the mouse functional genomics [34]. Moreover, the platform provides comprehensive phenotypes for mouse mutants, cataloging over 11 000 genes. After submitting proteins with positive colocalization, all relative system abnormalities have been parsed. For models that displayed psoriasis-related pathogenesis or manifestations, we reorganized them into two distinct groups associated with (i) immune abnormalities and (ii) skin abnormalities.

## Results

### Genome-wide meta-analysis results for psoriasis

**Figure 2:**
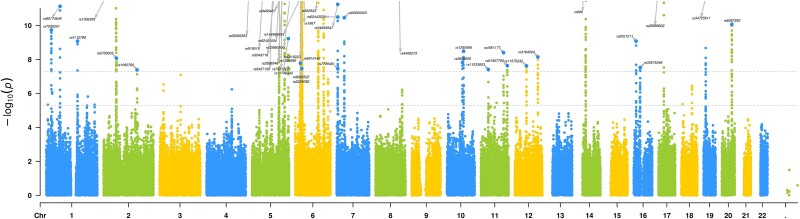
Manhattan plots for psoriasis in large-scale genome-wide association study meta-analysis. Genome-wide significance threshold (*P* = 5 × 10^−8^).

To increase the power of GWASs, meta-analysis is performed to increase sample size using Metal software [35, 36]; a meta-analysis of GWAS was performed on psoriasis from three nonintersecting databases to seek out the significant loci (*P* < 5 × 10^−8^) in the results (Supplementary Table S3). A total of 42 loci matched genome-wide significance; its Manhattan plot was shown in Fig. 2. Among these loci, nine loci overlapped with previous research, indicating the reliability of our analysis. Meanwhile, the other 33 loci were identified as newly discovered loci. The 33 loci reached the stricter threshold (*P* < 1 × 10^−9^), including 25 new loci such as rs74817271, rs583522, and rs9268522. The genomic inflation factor (λGC) was 1.014, and the LDSC intercept was 1.05 (se = 0.02, Supplement Table S4), indicating no genome inflation. In summary, genetic variation was identified at new loci for psoriasis and offered a more robust dataset for subsequent MR Analyses. To ensure the accuracy of our analysis, we utilized a combination of UKB and FinnGen as our discovery cohort while treating the GCST005527 dataset as an independent replication cohort. We detected 23 SNPs were replicated (Supplementary Table S5, Supplementary Fig. S1).

### Mendelian randomization reveals 19 proteins’ casual association with psoriasis using *cis*-protein quantitative trait loci

The study combined three pQTL data and conducted a two-sample MR analysis of the European population with psoriasis. Based on FDR values <0.05, 19 significant MR results for psoriasis were found during the process (Supplementary Table S6). Among them, eight proteins have the potential to decrease the risk of psoriasis. IL12B/IL23A (interleukin 12B/interleukin 23 subunit alpha) displayed the most significant association (OR = 0.714691103 [0.638068966, 0.800514363], FDR = 1.78E-06). In addition, 11 proteins were identified to increase the risk of psoriasis. Notably, IER3 (immediate early response 3) exhibited the most significant MR result (OR = 414.8566177 [239.0797123, 719.8687483], FDR = 9.19E-99), which was recognized for its regulatory influence on inflammation and immune system modulation, primarily through the NF-κB, PI3K/Akt, and MAPK/ERK signaling pathways [37]. Furthermore, this study marks the initial identification of several proteins—MAPRE1, SWAP70, VPS26A, BRD2, and B3GNT2—as potentially contributing factors in the pathogenesis of psoriasis. In our MR analysis, there was no significant heterogeneity and pleiotropy among the proteins. In addition, during the entire analysis process, any tool variables of protein were reserved for passing the *F*-value standard (*F* > 10).

### Colocalization analysis identifies six associated proteins for psoriasis

Colocalization analysis after positive MR results was required to be conducted and approved. Six high-evidence proteins among the above 19 proteins (PP.H4 > 0.9, Table 1) and three medium-colocalization results (0.9 > PP.H4 > 0.5, Supplementary Table S7) were found out. Among these proteins, AIF1, FCGR3A (Fc gamma receptor IIIa), and NEU1 (neuraminidase 1) were considered the strongest candidates for psoriasis risk (PP.H4 = 1). By the way, LocusZoom plots are shown in Supplementary Fig. S2.

**Table 1:** Mendelian randomization analysis and colocalization of six associated proteins with psoriasis

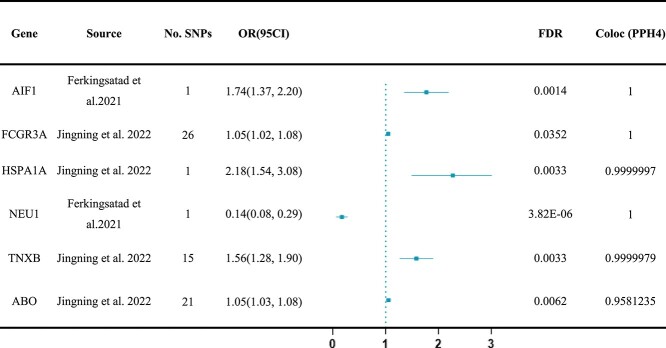

CI, confidence interval; FDR, false discovery rate; OR, odds ratio; No. SNPs, the number of single-nucleotide polymorphisms.

### Evaluation of druggability of associated proteins

The six associated proteins were then identified to investigate their development prospects. Drugs targeting several genes that were approved or in the clinical stage were listed (Supplementary Table S8). However, the drugs based on the screened six proteins have not yet been exploited for psoriasis treatment. Among them, two proteins have already been targeted by clinical drugs. In detail, drugs targeting FCGR3A were mainly used to bind aggregated IgG or monomeric IgG and then regulate cellular phagocytosis. Targeting NEU1, drugs participated in removing glycoprotein and sugar lipid sialic acid and were majorly used to regulate salivary amylase. Except for the above two proteins, drugs targeting the remaining four proteins have not been found, which needs further exploration.

**Figure 3:**
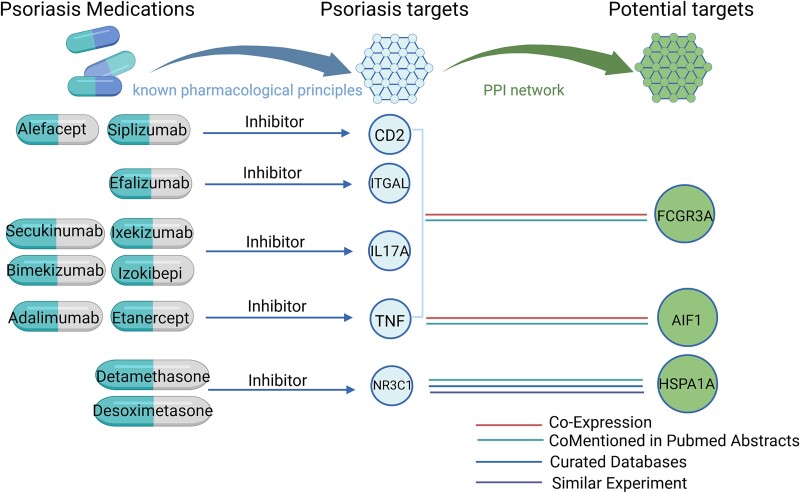
Interaction between current psoriasis medications targets and identified six associated proteins. PPI, protein–protein interaction. Figure was created with Biorender.com.

With this purpose, PPI network analysis was then conducted. The PPI network results revealed interactions between colocalization-positive proteins and existing therapeutic targets for psoriasis (Supplementary Table S9, Supplementary Fig. S3). Three potential targets had correlation scores >0.7 for known targets (Fig. 3). Specifically, FCGR3A showed a strong association with CD2, a known psoriatic target (Score 0.922). CD2 is the target of alefacept and siplizumab. The co-expression of these two targets was also revealed.

### Mouse knock-out models for potential proteins

To further predicate the pathological effects of these screened proteins on psoriasis, mouse knock-out (KO) models were then conducted using MGI resources. After analyzing evidence from KO models, we retrieved six psoriasis susceptibility genes, one gene for skin lesions, and one gene for both immune cell abnormalities and skin lesions. KO models on FCGR3A and ABO (alpha 1-3-*N*-acetylgalactosaminyltransferase and alpha 1-3-galactosyltransferase) showed phenotypes such as increased T-cell number, abnormal neutrophil physiology, and aberrant skin morphology suggesting a susceptible role in the pathogenesis of psoriasis (Supplementary Table S10).

## Discussion

In this study, a proteome-wide MR study was performed to uncover proteins that may be causally related to psoriasis and provide potentially promising therapeutic targets in the prevention or treatment of psoriasis. A GWAS meta-analysis was performed, and 33 possibly novel psoriasis susceptibility loci were found, providing a fresh GWAS data. Based on the GWAS data, further MR analysis and colocalization analysis genetically predicted that AIF1, FCGR3A, NEU1, HSPA1A, TNXB (Tenascin XB), and ABO were associated with psoriasis risk. Combined with the protein target druggability, mouse KO models, and PPI network, FCGR3A, AIF1, and HSPA1A were highlighted for functioning as potential targets for psoriasis with strong evidence. Together, this research provided novel targets for psoriasis treatment.

In order to explore the genetic architecture of psoriasis, we performed a genome-wide meta-analysis with available data from three independent cohorts, a total of 863 080 participants. Our meta-analysis of 20 105 cases and 842 975 controls identified 42 possible psoriasis susceptibility loci. Consistent with a previous study, we also identified nine susceptibility loci [38–41], which play an important role in the biological mechanism of psoriasis including skin barrier function and repair mechanisms [42], skin inflammation [43], development of psoriatic plaques and keratinocytes [44], and immune dysregulation [45]. Among the 33 novel psoriasis susceptibility loci, several genes have emerged as potential risk factors. TNFAIP3/rs583522, encoding the A20 protein, was significantly associated with psoriasis due to its role in down-regulating inflammation through the NF-kB pathway [46]. Other genes, NFKBIA/rs696 and FBXL19/rs35675346, also implicated in the NF-kB pathway, were predicted to be strongly associated with the disease [9, 47]. IL-13/rs1295686 was up-regulated in psoriatic lesions, altering the IL-13 cytokine and contributing to disease risk [48]. TYK2/rs34725611 and STAT2/rs11575232, part of the JAK-STAT pathway, are crucial for immune cell function and cytokine regulation [49, 50]. IL-23R/rs80174646, upon binding to IL-23, induces differentiation of naive T cells into Th17 and Th22 cells, leading to epidermal hyperplasia and inflammation [51]. ETS-1/rs6190776 5 promotes IFN-γ secretion and Th1/Th17 cell differentiation, potentially driving psoriasis [52]. Furthermore, we conducted meta-analyses using UKB and FinnGen data, followed by independent validation with a third dataset. The meta-analysis identified that 23 of 31 SNPs were validated. These unvalidated SNPs, as well as the 42 SNPs that were significant in our original analysis, showed significant association in the raw data (*P* < 5e-8). However, they were not identified as genome-wide significant SNPs in the original datasets due to LD clumping. After our meta-analysis, the weight of different SNPs was recalculated, allowing for the selection of lead SNPs. Our analysis initially focused on SNPs that appeared at least twice across the three datasets, thus supporting their association with psoriasis.

In current research, FCGR3A was identified as a potential target for psoriasis with robust evidence. FCGR3A belongs to low affinity located ~82 kb of two repeated sections of FCGR gene families [53]. A previous study has discovered that the FCGR3A-V158F polymorphism was associated with the treatment of psoriasis patients [54]. Furthermore, the study provided profound evidence for the role of FCGR3A in the pathogenesis of psoriasis. Mechanistically, Fc gamma receptors are expressed on immune cells such as monocytes and macrophages. By binding to IgG, FCGR3A participates in the induction and regulation of inflammatory processes, leading to the release of proinflammatory cytokines and other tissue-destructive mediators from activated monocytes/macrophages, which may contribute to the development of psoriasis [55]. In our druggability analysis, drugs targeting FCGR3A such as cetuximab, etanercept, and alemtuzumab have been clinically approved. Moreover, FCGR3A was found to interact with known therapeutic targets of psoriasis such as CD2, Tumor necrosis factor (TNF), Integrin subunit alpha L (ITGAL), and IL17A. For example, the alteration of FCGR3A gene expression can cause nonresponse to anti-TNF therapy in patients with inflammatory diseases [56]. In the FCGR3A KO model, the number of activated T cells and the function of macrophages were altered.

AIF1, another potential target of psoriasis, is a Ca^2+^-binding EF-hand cytokine encoded in the major histocompatibility complex III region on chromosome 6. AIF1 is expressed in inflammatory cells and a regulated cell state [57]. It was revealed that AIF1 can induce the expression of type I collagen, type III collagen, and cytokines by skin fibroblasts and also promote the production of THF-a and other cytokines in T cells and macrophages [58]. A previous study showed that AIF1 was upregulated in psoriasis patients [59]. Consistent with published findings, our study provided further evidence that AIF1 was associated with psoriasis. In the PPI network analysis, TNF, a known target of psoriasis, is strongly correlated with AIF1. In *in vitro* and *in vivo* studies, the up-regulation of AIF1 induced by high glucose can promote the production of TNF-α, which causes inflammation [60]. Together, AIF1 functions as one reliable target for psoriasis.

In addition, HSPA1A has been verified as another target in psoriasis. HSPA1A is located in the histocompatibility complex III region and encodes a highly conserved 70-kDa heat-shock protein, HSP70. The roles of HSP70 in the pathogenesis of inflammation have been repeatedly incriminated [61]. In detail, HSP70 can directly activate inflammatory responses and host innate immunity by binding to TLR2 and TLR4 receptors through signaling pathways mediated by myeloid differentiation factor [61]. Simultaneously, the activation of the myeloid differentiation factor can activate mitogen-activated protein kinases and nuclear factor kappa-B, further promoting the inflammatory process [62]. Although the association between psoriasis and HSPA1A has not been reported, our findings suggest that HSPA1A may be a potential therapeutic target for psoriasis for the following reasons. Firstly, in the colocalization results, the PP.H4 values of HSPA1A in two different pQTL data [22, 23] were >0.9. Besides, our PPI network showed that HSPA1A has a significant correlation with NR3C1, a known target of psoriasis. Together, HSPA1A was another potential target because of its role in regulating HSP70 expression and data from colocalization results and PPI network analysis.

According to this study, NEU1, TNXB, and ABO were also related to psoriasis risk. NEU1 belongs to an ancient enzyme family of removing terminal sialic acids from glycoproteins and glycolipids. NEU1 has been shown to be involved in the regulation of immune cells. In addition to being activated during monocyte differentiation, NEU1 expression is also upregulated following the activation of neutrophils and T cells [63]. Drugs that target NEU1, such as oseltamivir, acetylsalicylic acid, and celecoxib, are currently available in clinical practice. Meanwhile, TNXB, a vital component of synovium, was significantly expressed in rheumatoid arthritis synovium by immunofluorescence labeling, showing that inflammation can promote TNXB production [64]. Moreover, the functional alleles A and B in the ABO locus respectively encode two glycosyltransferases, α-1-3-*N*-acetylgalactosamine transferase and α-1-3-galactosamine transferase [65]. It has been found that there is a correlation between psoriasis and different blood groups. The initiation and aggravation factors of psoriasis in different blood groups are significantly different. In the ABO KO model, skin morphology was altered. Although these three associated genes passed the colocalization test, they were not validated by the PPI network of psoriasis. Therefore, more research was needed to verify whether these three genes can be therapeutic targets for psoriasis.

The current study has several strengths. Firstly, this research integrated three well-known GWAS databases from the European population by using the meta-analysis approach and screened out the GWAS data with high quality and large sample size. Secondly, the subsequent validation of the colocalization-positive targets in a variety of ways increased the conviction, and thus, several key potential targets were obtained.

Even so, limitations also existed. The research population consisted of only European individuals, which limits the generalizability of conclusions to other populations. Besides, due to the inherent limitations of GWAS data, we were not able to categorize factors that might be associated with differences in gender, age, and lifestyle habits.

## Conclusion

In conclusion, the present research has found a causal relationship between circulating proteins and psoriasis risk through comprehensive analyses, providing new targets for future therapeutic strategies. AIF1, FCGR3A, NEU1, HSPA1A, TNXB, and ABO were such associated proteins. Among them, FCGR3A, AIF1, and HSPA1A are more promising.

**Key Points**

- The efficacy of drug therapy for psoriasis is not ideal enough, and more drugs are required to be developed.
- Three well-known GWAS databases from the European population were integrated by using the meta-analysis approach and screened out the GWAS data with high quality and large sample size.
- Mendelian randomization and colocalization analysis on the large-scale GWAS data were performed to identify gene-validated drug targets.

## Supplementary Material

Supplementary_Figure_bbaf032

Supplementary_table_bbaf032

## Data Availability

The original database used and summary results in the study are included in the article/Additional file. GWAS summary statistics for psoriasis from the FinnGen consortium: https://storage.googleapis.com/finngen-public-data-r9/summary_stats/finngen_R9_L12_PSORIASIS.gz. GWAS catalog data: https://www.ebi.ac.uk/gwas/. UKB: https://yanglab.westlake.edu.cn/data/ukb_fastgwa/imp_binary/. R package “TwoSampleMR”: https://mrcieu.github.io/TwoSampleMR. R package “coloc”: http://cran.r-project.org/web/packages/coloc. STRING database: https://string-db.org/. MGI: http://www. informatics.jax.org/. Locuscompare: https://github.com/boxiangliu/locuscomparer.
