# Supplementary material for: Novel genetic insight for psoriasis: integrative genome-wide analyses in 863 080 individuals and proteome-wide Mendelian randomization
Source: Brief Bioinform. 2025 Jan 30;26(1):bbaf032. doi: 10.1093/bib/bbaf032 (PMC11781221; doi:10.1093/bib/bbaf032)
Supplement: Supplementary_Figure_bbaf032 [file supplementary_figure_bbaf032.docx]

**Novel genetic insight for Psoriasis: Integrative Genome-Wide Analyses in 863,080 Individuals and Proteome-Wide Mendelian Randomization**

**Supplementary Figure S1. Thirty-one genetic loci associated with psoriasis.**

**Supplementary Figure S2：LocusZoom plots of colocalization results**. A-K, the pQTL data was

derived from Jingning et al. 2022; L-P, the pQTL data source was from Ferkingsatad et al.2021; Q- S, was obtained from Sun et al.2018 . The dashed grey line represents the genome-wide significance threshold of 5x10-08. Neighboring genes in are shown at the bottom of the figure.

**Supplementary Figure S3: protein-protein interaction network**. Results revealed interactions between colocalization positive proteins and existing therapeutic targets for psoriasis (scores greater than 0.7).

**Supplementary Figure S1.**


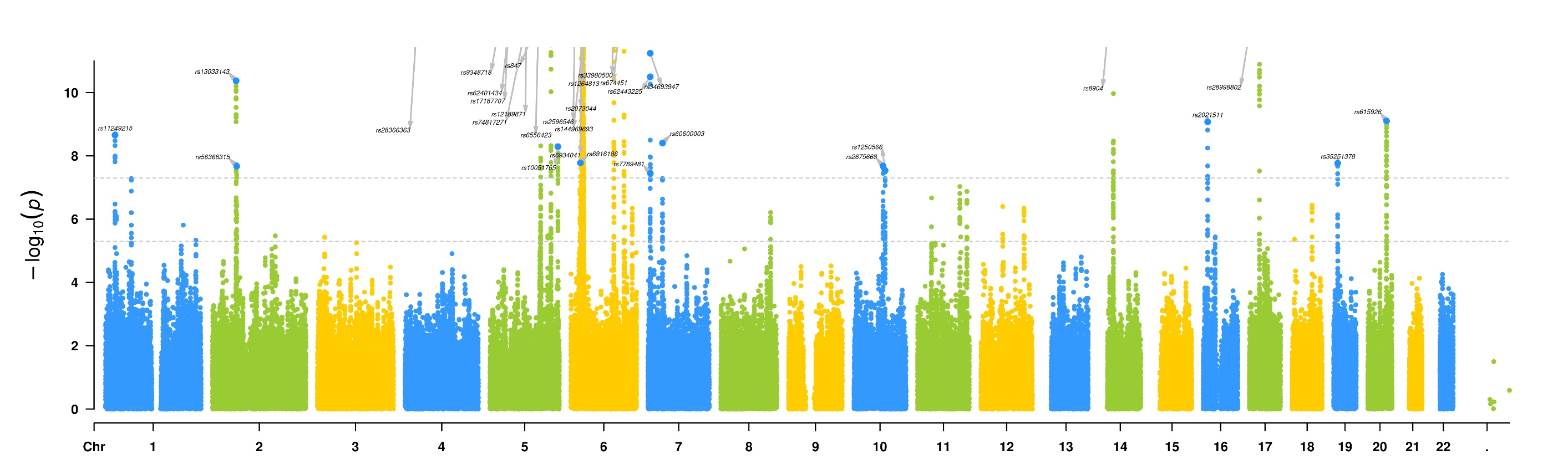


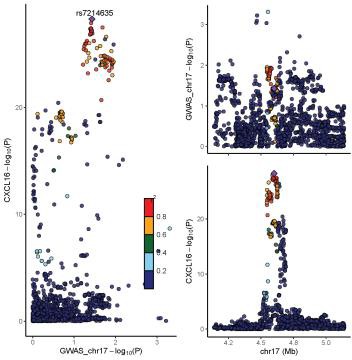

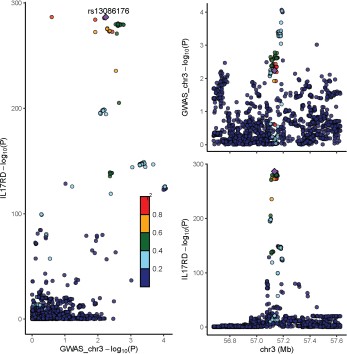

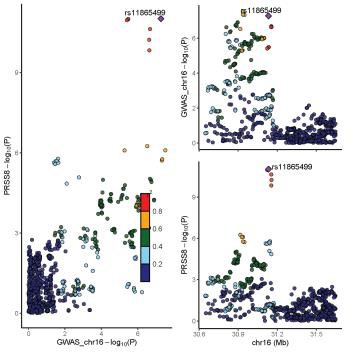

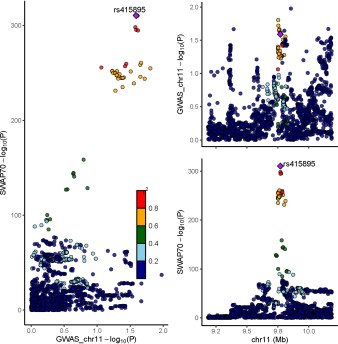

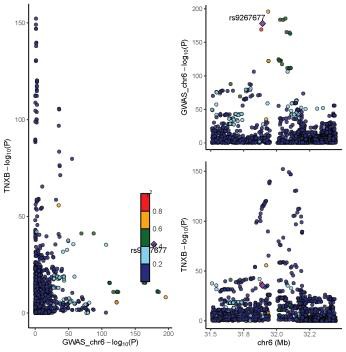

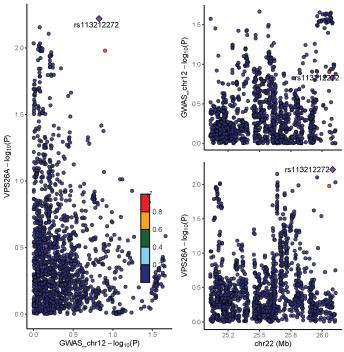
A B C D E F


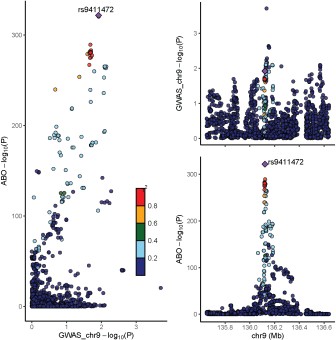

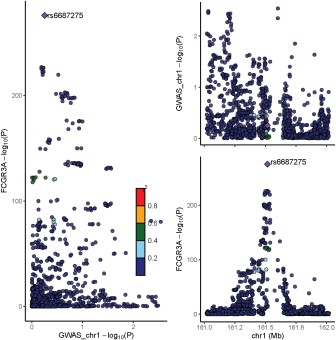

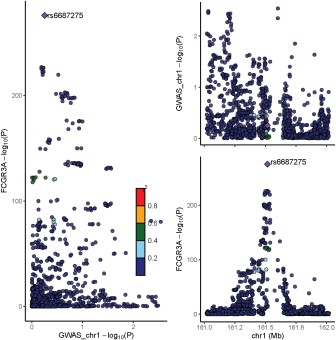

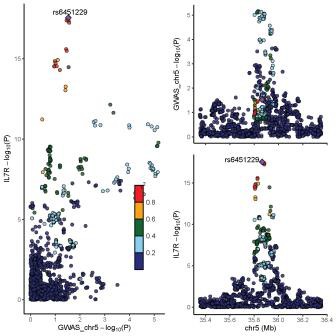

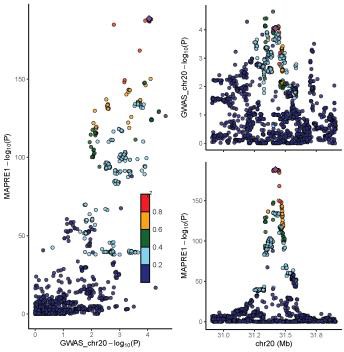

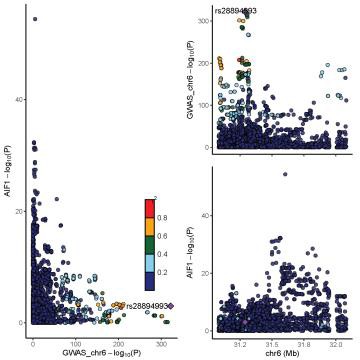
G H I J K L


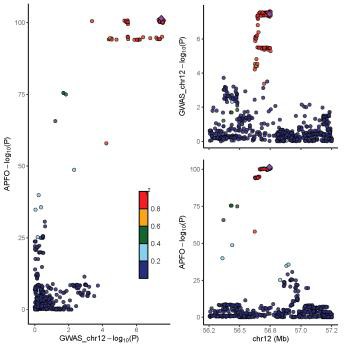

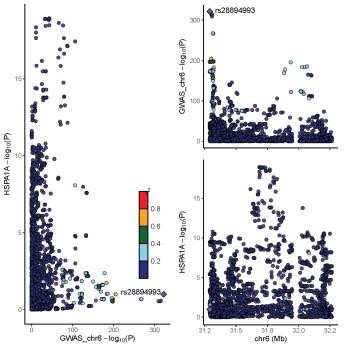

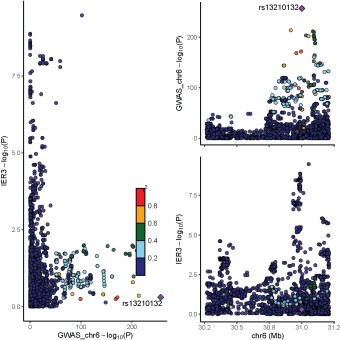

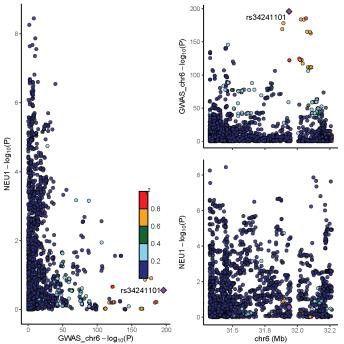

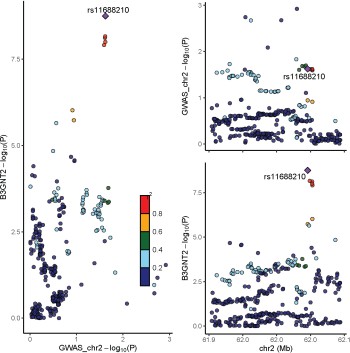

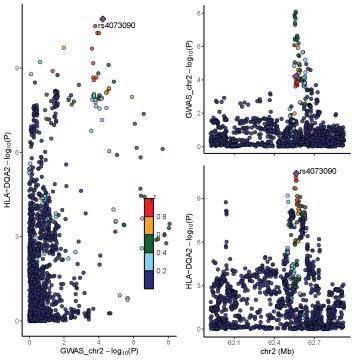
M N O P Q R


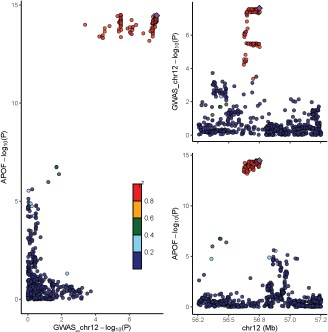
S

**Supplementary Figure S2**


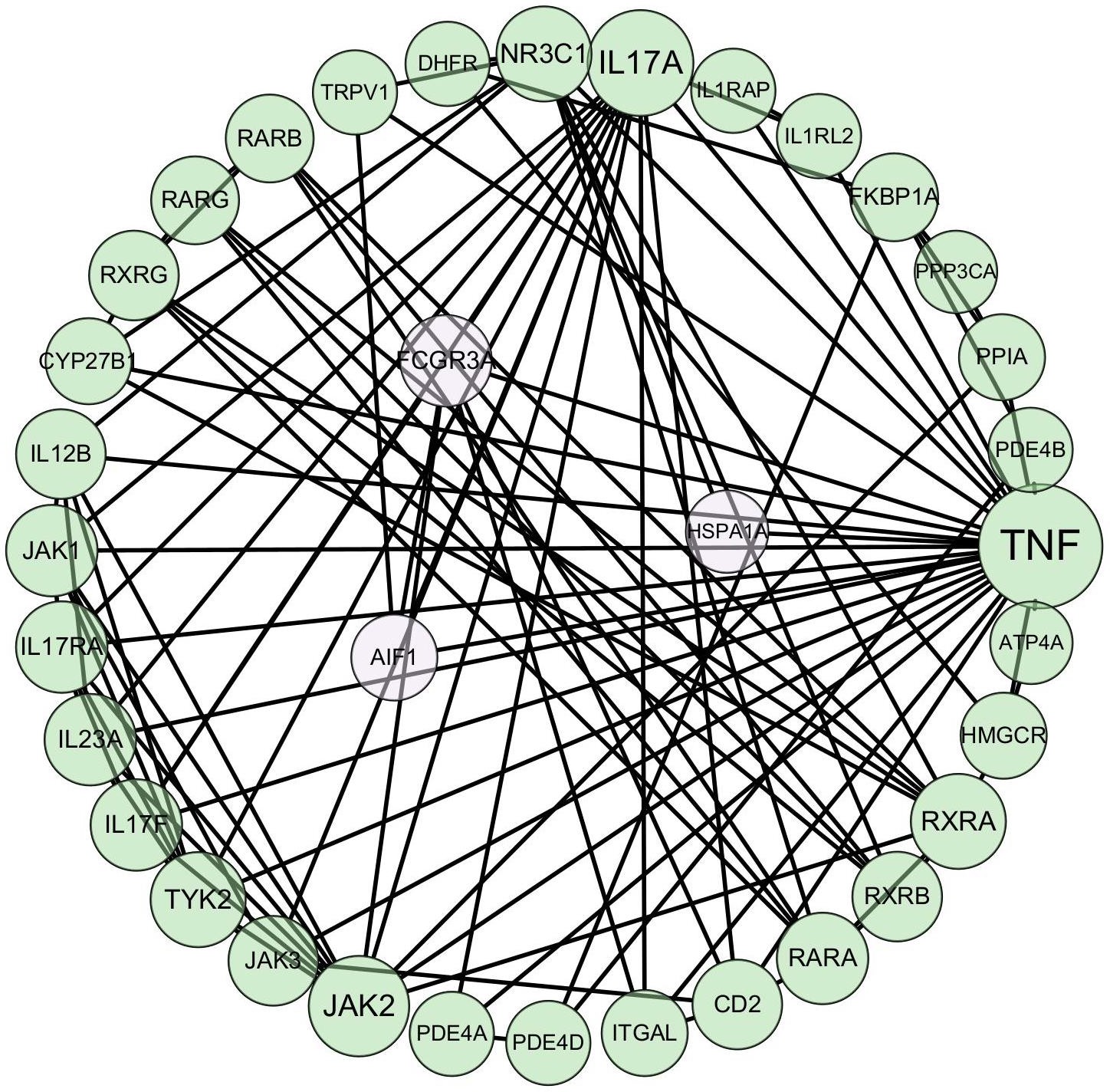


**Supplementary Figure S3**
