# Supplementary material for: Novel genetic insight for psoriasis: integrative genome-wide analyses in 863 080 individuals and proteome-wide Mendelian randomization
Source: Brief Bioinform. 2025 Jan 30;26(1):bbaf032. doi: 10.1093/bib/bbaf032 (PMC11781221; doi:10.1093/bib/bbaf032)
Supplement: Supplementary_table_bbaf032 [file supplementary_table_bbaf032.docx]

| **Supplementary Table S1: Summary of proteins datasets used in the current study.** | | | |  |  | |  |  |  |  |  |  |
| --- | --- | --- | --- | --- | --- | --- | --- | --- | --- | --- | --- | --- |
| **Supplementary Table S2.** **Summary of GWAS dataset used in the current study.** | | | | | | | | | | | | |
| **Supplementary Table S3. Forty-two genetic loci associated with psoriasis.** | | |  |  |  | |  |  |  |  |  |  |
| **Supplementary Table S4. Genomic inflation (lambda), LDSC intercept, intercept standard error, and sample size for each dataset.** | | | | | | | | | |  |  |  |
| **Supplementary Table S5. Thirty-one genetic loci associated with psoriasis using FinnGen and UK Biobank.** | | | | | |  |  |  |  |  |  |  |
| **Supplementary Table S6. Mendelian randomization results.** | |  |  |  |  | |  |  |  |  |  |  |
| **Supplementary Table S7. Colocalization results.** |  |  |  |  |  | |  |  |  |  |  |  |
| **Supplementary Table S8. Druggability results in the drugbank.** | |  |  |  |  | |  |  |  |  |  |  |
| **Supplementary Table S9. PPI network results between psoriasis-related proteins and mature target proteins.**  **Supplementary Table S10. Effects after knocking out psoriasis-relative genes.** | | | | | | | |  |  |  |  |  |

| Supplementary Table 1: Summary of proteins datasets used in the current study. | | | | | |
| --- | --- | --- | --- | --- | --- |
| Study | Number of proteins | Number of pQTLs | Sample size | Measure Platform | PMID |
| Ferkingsatad et al.2021 | 4,719 | 18,084 | 35,559 | Somal ogic | 34857953 |
| Sun_et al.2018 | 2,995 | 1,927 | 3,301 | Somal ogic | 29875488 |
| Jingning et al 2022 | 4,907 | 2,004 | 7,213 | SOMAmer | 35501419 |
| pQTL, cis-protein quantitative trait loci | | | | | |

| Supplementary Table 2. Summary of GWAS dataset used in the current study. | | | | | | | | | | | | | | | | |
| --- | --- | --- | --- | --- | --- | --- | --- | --- | --- | --- | --- | --- | --- | --- | --- | --- |
| Study | Contry | Genome build | lambda | Platform | SNP level exclusions | | | Subject level exclusions | | Imputation software and reference sample | | Statistical analysis | | | | |
|  |  |  |  |  | MAF | Genotype call rate (%) | HWE P-value | Sample call rate (%) | Other exclusions | Software | Reference panel | Software | Cases | Controls | Sample size | Statistical Model |
| FinnGen | Finland | 38 | 1.1651 |  | <0.0001 | <95 | 1 x 10-9 | < 97 | high genotype missingness (>5%) and excess heterozygosity (±4 standard deviations) were removed. In variant-wise QC,  variants with high missingness (>2%) and minor allele count < 3 were removed | BWA-MEM | Sequencing Initiative Suomi (SISu) v.3 | PLINK | 8075 | 330975 | 339050 | Logistic |
| UK Biobank | UK | 37 | 1.0046 | UK BiLEVE Axiom Array/ UK Biobank Axiom Array | <0.0001 | For further details, please see Bycroft C et al. PMID: 30305743 | | | genotyping rate ≥0.9 and imputation info score ≥0.8 | IMPUTE4 | the Haplotype Reference Consortium and the UK10K project | PLINK | 250 | 456098 | 456348 | Logistic |
| LAM* | Canada, U.S., Austria, et al | 37 | 1.3581 | Illumina | <0.05 | <95 | <1 x 10-6 | <98 | SNPs with low imputation quality (r2 ≤ 0.3 for minimac and info score < 0.5 for IMPUTE2) were removed | minimac and IMPUTE2 | the 1000 Genomes Project and alignment release | PLINK | 10588 | 22806 | 33394 | Logistic |
| MAF, minor allele frequency; HWE, Hardy-Weinberg Equilibrium; *LAM dataset is a meta-analysis of 3 genome-wide association studies and 2 independent databases | | | | | | | | | | | | | | | | |

| Supplementary Table 3. Forty-two genetic loci associated with psoriasis | | | | | | | | | | | |
| --- | --- | --- | --- | --- | --- | --- | --- | --- | --- | --- | --- |
| SNP | Chromosome | Positiona | Geneb | Known locus | EA | NEA | EAF | Meta-analysis | | | |
|  |  |  |  |  |  |  |  | Beta | SE | P | Direction |
| rs11033603 | 11 | 36434542 | PRR5L | Novel | a | g | 0.9418231 | 0.1741 | 0.0317 | 3.91E-08 | ++? |
| rs11575232 | 12 | 56744422 | STAT2 | Novel | a | g | 0.9454161 | -0.1845 | 0.0331 | 2.40E-08 | --- |
| rs11746443 | 5 | 176798306 | RGS14 | Novel | a | g | 0.668291 | 0.0884 | 0.0143 | 5.93E-10 | +++ |
| rs1250566 | 10 | 81046453 | ZMIZ1 | Novel | a | g | 0.592224 | -0.0904 | 0.0153 | 3.31E-09 | --- |
| rs1295686 | 5 | 131995843 | IL13 | Novel | t | c | 0.369195 | -0.1243 | 0.0161 | 1.34E-14 | --+ |
| rs1306395 | 2 | 61076272 | REL-DT | Novel | t | c | 0.587646 | 0.1106 | 0.0149 | 1.18E-13 | ++- |
| rs144969693 | 6 | 33566867 |  | Novel | t | c | 0.9317919 | 0.2133 | 0.0301 | 1.35E-12 | +?+ |
| rs1967 | 6 | 30125537 | TRIM10 | Novel | t | c | 0.9634333 | 0.5125 | 0.0303 | 2.47E-64 | +++ |
| rs1990760 | 2 | 163124051 | IFIH1 | Know | t | c | 0.414216 | 0.0832 | 0.0152 | 4.15E-08 | +++ |
| rs2021511 | 16 | 11344903 | LOC105371082 | Novel | t | c | 0.734839 | -0.1127 | 0.0184 | 8.41E-10 | -?- |
| rs2229092 | 6 | 31540757 | LTA LOC100287329 | Novel | a | c | 0.9459573 | 0.2044 | 0.0371 | 3.44E-08 | +?+ |
| rs2596546 | 6 | 31329386 |  | Novel | a | g | 0.65296 | -0.1863 | 0.0164 | 8.74E-30 | -?- |
| rs2688608 | 10 | 75658349 |  | Novel | t | g | 0.530295 | 0.0833 | 0.0147 | 1.50E-08 | +++ |
| rs28366363 | 6 | 32565355 |  | Novel | a | g | 0.9437937 | 0.5835 | 0.0287 | 1.16E-91 | +?+ |
| rs28998802 | 17 | 26124908 | NOS2 | Know | a | g | 0.815746 | 0.1551 | 0.0187 | 9.31E-17 | ++- |
| rs3184504 | 12 | 111884608 | SH2B3 | Know | t | c | 0.408452 | 0.0864 | 0.0149 | 7.17E-09 | ++- |
| rs33980500 | 6 | 111913262 | TRAF3IP2 TRAF3IP2-AS1 | Know | t | c | 0.9275512 | 0.278 | 0.0259 | 8.53E-27 | +++ |
| rs34693947 | 7 | 5427517 | TNRC18 | Novel | a | g | 0.9015469 | 0.1818 | 0.0264 | 5.73E-12 | +?+ |
| rs34725611 | 19 | 10477067 | TYK2 | Novel | a | g | 0.743747 | 0.117 | 0.0167 | 2.23E-12 | +++ |
| rs35675346 | 16 | 30936081 | FBXL19 FBXL19-AS1 | Novel | a | g | 0.729281 | 0.0904 | 0.0163 | 3.03E-08 | +++ |
| rs4112788 | 1 | 152551276 | LCE | Know | a | g | 0.337961 | -0.0965 | 0.0157 | 8.41E-10 | --- |
| rs4406273 | 6 | 31266090 | LINC02571 LOC112267902 | Know | a | g | 0.9389662 | 0.9422 | 0.0235 | 0.00E+00 | +++ |
| rs4561177 | 11 | 109962432 | ZC3H12C | Know | a | g | 0.66752 | 0.0931 | 0.0158 | 4.00E-09 | ++- |
| rs583522 | 6 | 138189884 | TNFAIP3 WAKMAR2 | Novel | t | c | 0.333478 | -0.1328 | 0.0157 | 2.26E-17 | --- |
| rs60600003 | 7 | 37382465 | ELMO1 | Novel | t | g | 0.897896 | -0.1578 | 0.0238 | 3.52E-11 | --- |
| rs6067293 | 20 | 48581741 |  | Novel | t | c | 0.419781 | 0.0989 | 0.0152 | 8.56E-11 | +++ |
| rs61907765 | 11 | 128391937 | ETS1 MIR6090 | Novel | t | c | 0.767034 | 0.0958 | 0.0172 | 2.32E-08 | +++ |
| rs62401434 | 6 | 28065038 |  | Novel | a | g | 0.9533463 | 0.4077 | 0.0336 | 8.20E-34 | +?+ |
| rs62443225 | 7 | 5482137 |  | Novel | a | g | 0.9219981 | 0.1907 | 0.0287 | 3.17E-11 | +?- |
| rs6457109 | 6 | 29933261 |  | Novel | t | c | 0.9265085 | -0.3996 | 0.0249 | 7.63E-58 | --- |
| rs6759003 | 2 | 62559205 |  | Novel | t | c | 0.33841 | 0.0915 | 0.0159 | 8.43E-09 | +++ |
| rs6916186 | 6 | 24708523 | C6orf62 | Novel | a | g | 0.086101 | 0.1566 | 0.0278 | 1.67E-08 | +?+ |
| rs6918223 | 6 | 32722532 |  | Novel | a | c | 0.44874 | -0.1459 | 0.0143 | 2.23E-24 | --- |
| rs696 | 14 | 35871093 | NFKBIA | Novel | t | c | 0.611163 | -0.1221 | 0.0154 | 2.55E-15 | --- |
| rs74817271 | 5 | 150469973 |  | Know | a | g | 0.9255618 | 0.2628 | 0.0267 | 6.74E-23 | +++ |
| rs7536201 | 1 | 25293084 | RUNX3 | Know | t | c | 0.448999 | -0.0963 | 0.0151 | 1.86E-10 | --- |
| rs7789481 | 7 | 5793570 | RNF216 | Novel | c | g | 0.86997 | 0.1278 | 0.0232 | 3.55E-08 | +?- |
| rs80174646 | 1 | 67708155 | IL23R | Novel | t | g | 0.954284 | -0.2546 | 0.0372 | 7.43E-12 | --- |
| rs918518 | 5 | 158826493 |  | Novel | a | g | 0.203532 | 0.2167 | 0.0177 | 1.69E-34 | +++ |
| rs9268522 | 6 | 32381443 |  | Novel | a | t | 0.813585 | -0.1648 | 0.0197 | 6.40E-17 | -?- |
| rs9348718 | 6 | 26456280 | BTN2A1 | Novel | a | g | 0.9621425 | -0.3999 | 0.037 | 2.91E-27 | -?- |
| rs9468487 | 6 | 29107052 |  | Novel | a | g | 0.9619941 | -0.2645 | 0.0337 | 3.82E-15 | --- |
| SNP, single nucleotide polymorphism; EA, effect allele; NEA, non-effect allele; EAF, effect allele frequency; SE, standard error; | | | | | | | | | | | |

| Dataset | Lambda | Intercept | Intercept standard error | N |
| --- | --- | --- | --- | --- |
| FinnGen | 1.1651 | 1.0662 | 0.0103 | 339050 |
| UK Biobank | 1.0046 | 0.986 | 0.0069 | 456348 |
| LAM* | 1.3581 | 1.1697 | 0.0343 | 33394 |
| Meta-analysis | 1.014 | 1.05 | 0.02 | 828792 |

Supplementary Table 4. Genomic inflation (lambda), LDSC intercept, intercept standard error, and sample size for each dataset

| Supplementary Table S5. Thirty-one genetic loci associated with psoriasis using FinnGen and UK Biobank. | | | | | | | | | | | | |  |  | | | | | | |  | | | | | | |  |
| --- | --- | --- | --- | --- | --- | --- | --- | --- | --- | --- | --- | --- | --- | --- | --- | --- | --- | --- | --- | --- | --- | --- | --- | --- | --- | --- | --- | --- |
| SNP | Chromosome | Position | EA | NEA | Beta | SE | P value | Direction | EAF | | Duplication | | | Ldproxy | R2 | Chromosome | | Position | Freq | | | | | P value | |  |  |  |
| rs10051765 | 5 | 176799992 | t | c | -0.0945 | 0.0162 | 5.10E-09 | -- | 0.598493 | | |  | | rs11746443 | 7.64E-01 | 5.00E+00 | | 1.77E+08 | | 1.77E-01 | | | 1.03E-03 | |  |  |  |  |
| rs11249215 | 1 | 25297184 | a | g | 0.0957 | 0.016 | 2.19E-09 | ++ | 0.462682 | |  | | | rs10751775 | 9.08E-01 | 1.00E+00 | | 2.53E+07 | | 6.77E-01 | | | 5.37E-03 | |  |  |  |  |
| rs12189871 | 6 | 31251924 | t | c | 0.8133 | 0.0264 | 1.28E-208 | ++ | 0.9389219 | | √ | | |  |  |  | |  | |  | | |  | |  |  |  |  |
| rs1250566 | 10 | 81046453 | a | g | -0.0909 | 0.0164 | 2.90E-08 | -- | 0.592224 | | √ | | |  |  |  | |  | |  | | |  | |  |  |  |  |
| rs1264813 | 6 | 29939900 | t | c | -0.1682 | 0.0203 | 1.37E-16 | -- | 0.790764 | |  | | | rs1150743 | 9.46E-01 | 6.00E+00 | | 3.00E+07 | | 1.35E-01 | | | 6.56E-09 | |  |  |  |  |
| rs13033143 | 2 | 61161095 | a | g | 0.1203 | 0.0182 | 4.22E-11 | +- | 0.75446 | |  | | | rs9309331 | 1.00E+00 | 2.00E+00 | | 6.11E+07 | | 1.50E-01 | | | 2.82E-04 | |  |  |  |  |
| rs144969693 | 6 | 33566867 | t | c | 0.2133 | 0.0301 | 1.35E-12 | ++ | 0.9317919 | | \ | | |  |  |  | |  | |  | | |  | |  |  |  |  |
| rs17187707 | 6 | 30036528 | a | g | -0.5568 | 0.0345 | 1.39E-58 | -- | 0.9603877 | | √ | | |  |  |  | |  | |  | | |  | |  |  |  |  |
| rs2021511 | 16 | 11344903 | t | c | -0.1127 | 0.0184 | 8.41E-10 | -- | 0.734839 |  | | | | rs413024 | 0.9148 | 16 | | 11354091 | | 0.221446 | | | 0.028333617 | |  |  |  |  |
| rs2073044 | 6 | 32338986 | t | c | 0.1476 | 0.0187 | 3.41E-15 | ++ | 0.782215 | √ | | | |  |  |  | |  | |  | | |  | |  |  |  |  |
| rs2596546 | 6 | 31329386 | a | g | -0.1863 | 0.0164 | 8.74E-30 | -- | 0.65296 | \ | | | |  |  |  | |  | |  | | |  | |  |  |  |  |
| rs2675668 | 10 | 75636148 | a | g | 0.0894 | 0.016 | 2.09E-08 | ++ | 0.548008 |  | | | | rs2688608 | 0.924 | 10 | | 75658349 | | 0.705671 | | | 0.001583014 | |  |  |  |  |
| rs28366363 | 6 | 32565355 | a | g | 0.5835 | 0.0287 | 1.16E-91 | ++ | 0.9437937 |  | | | | rs2856705 | 0.5888 | 6 | | 32670956 | | 0.153554 | | | 4.93096E-18 | |  |  |  |  |
| rs28998802 | 17 | 26124908 | a | g | 0.1542 | 0.02 | 1.36E-14 | +- | 0.815746 | √ | | | |  |  |  | |  | |  | | |  | |  |  |  |  |
| rs33980500 | 6 | 111913262 | t | c | 0.2429 | 0.0287 | 2.60E-17 | ++ | 0.9275512 |  | | | | rs33980500 | 1 | 6 | | 111913262 | | 0.0836661 | | | 3.64425E-19 | |  |  |  |  |
| rs34693947 | 7 | 5427517 | a | g | 0.1818 | 0.0264 | 5.73E-12 | ++ | 0.9015469 | \ | | | |  |  |  | |  | |  | | |  | |  |  |  |  |
| rs35251378 | 19 | 10459969 | a | g | -0.1034 | 0.0183 | 1.71E-08 | -- | 0.736424 |  | | | | rs34725611 | 0.9401 | 19 | | 10477067 | | 0.269369 | | | 2.76613E-09 | |  |  |  |  |
| rs56368315 | 2 | 62558512 | t | g | -0.0908 | 0.0162 | 2.12E-08 | -- | 0.403046 |  | | | | rs11687879 | 0.7754 | 2 | | 62550524 | | 0.508387 | | | 0.000363372 | |  |  |  |  |
| rs60600003 | 7 | 37382465 | t | g | -0.149 | 0.0253 | 3.93E-09 | -- | 0.897896 | √ | | | |  |  |  |  | | |  | | |  | |  |  |  |  |
| rs615926 | 20 | 48519258 | t | c | 0.0987 | 0.0161 | 7.87E-10 | ++ | 0.420215 |  | | | | rs4810997 | 0.9795 | 20 | 48579348 | | | 0.46246 | | | 0.005006961 | |  |  |  |  |
| rs62401434 | 6 | 28065038 | a | g | 0.4077 | 0.0336 | 8.20E-34 | ++ | 0.9533463 | \ | | | |  |  |  |  | | |  | | |  | |  |  |  |  |
| rs62443225 | 7 | 5482137 | a | g | 0.1907 | 0.0287 | 3.17E-11 | +- | 0.9219981 | \ | | | |  |  |  |  | | |  | | |  | |  |  |  |  |
| rs6556423 | 5 | 158857189 | t | c | -0.1839 | 0.0163 | 1.85E-29 | -- | 0.358228 |  | | | | rs4921496 | 0.6228 | 5 | 158848071 | | | 0.183107 | | | 7.12657E-09 | |  |  |  |  |
| rs674451 | 6 | 138216788 | t | c | -0.125 | 0.0165 | 4.17E-14 | -- | 0.655687 | √ | | | |  |  |  |  | | |  | | |  | |  |  |  |  |
| rs6916186 | 6 | 24708523 | a | g | 0.1566 | 0.0278 | 1.67E-08 | ++ | 0.086101 | \ | | | |  |  |  |  | | |  | | |  | |  |  |  |  |
| rs6934041 | 6 | 32722767 | c | g | -0.1388 | 0.016 | 4.37E-18 | -- | 0.448114 |  | | | | rs10807113 | 0.992 | 6 | 32722186 | | | 0.57488 | | | 2.331E-12 | |  |  |  |  |
| rs74817271 | 5 | 150469973 | a | g | 0.2349 | 0.0288 | 3.08E-16 | ++ | 0.9255618 | √ | | | |  |  |  |  | | |  | | |  | |  |  |  |  |
| rs7789481 | 7 | 5793570 | c | g | 0.1278 | 0.0232 | 3.55E-08 | +- | 0.86997 | \ | | | |  |  |  |  | | |  | | |  | |  |  |  |  |
| rs847 | 5 | 131996669 | t | c | -0.1227 | 0.0169 | 3.82E-13 | -+ | 0.36501 |  | | | | rs1295685 | 0.994 | 5 | 131996445 | | | 0.228035 | | | 0.000186451 | |  |  |  |  |
| rs8904 | 14 | 35871217 | a | g | -0.1265 | 0.0165 | 1.51E-14 | -- | 0.611941 | √ | | | |  |  |  |  | | |  | | |  | |  |  |  |  |
| rs9348718 | 6 | 26456280 | a | g | -0.3999 | 0.037 | 2.91E-27 | -- | 0.9621425 | \ | | | |  |  |  |  | | |  | |  | | |  |  |  |  |
| SNP, single nucleotide polymorphism; EA, effect allele; NEA, non-effect allele; EAF, effect allele frequency; SE, standard error; Freq, frequency; Plus sign (+) indicates a positive direction of effect and a minus sign (-) indicates a negative direction of effect. Question mark means that the variant is missing in the specific cohort. | | | | | | | | | | | | | | | | | | | | | | | | | |  |  |  |
|  |  |  |  |  |  |  |  |  |  |  |  |  |  |  |  |  |  |  |  |  |  |  |  |  |  |  |  |  |
|  |  |  |  |  |  |  |  |  |  |  |  |  |  |  |  |  |  |  |  |  |  |  |  |  |  |  |  |  |
|  |  |  |  |  |  |  |  |  |  |  |  |  |  |  |  |  |  |  |  |  |  |  |  |  |  |  |  |  |

| Supplementary Table 6. Mendelian randomization results | | | | | | | | | | | | |
| --- | --- | --- | --- | --- | --- | --- | --- | --- | --- | --- | --- | --- |
| exposure | outcome | method | nsnp | b | se | P value | or | or_lci95 | or_uci95 | fdr | source |  |
| PRSS8 | psoriasis | Wald ratio | 1 | 0.671593 | 0.124221 | 6.43E-08 | 1.957352 | 1.534371 | 2.496938 | 8.12E-05 | Jingning et al. 2022 |  |
| HSPA1A | psoriasis | Wald ratio | 1 | 0.779034 | 0.176914 | 1.07E-05 | 2.179367 | 1.540772 | 3.082637 | 0.003285 | Jingning et al. 2022 |  |
| IL7R | psoriasis | Wald ratio | 1 | 0.617718 | 0.166872 | 0.000214 | 1.85469 | 1.337296 | 2.572263 | 0.033949 | Jingning et al. 2022 |  |
| MAPRE1 | psoriasis | Inverse variance weighted | 14 | -0.12596 | 0.023689 | 1.05E-07 | 0.881648 | 0.841649 | 0.923548 | 8.12E-05 | Jingning et al. 2022 |  |
| SWAP70 | psoriasis | Inverse variance weighted | 16 | -0.06964 | 0.018847 | 0.00022 | 0.932734 | 0.898906 | 0.967834 | 0.033949 | Jingning et al. 2022 |  |
| FCGR3A | psoriasis | Inverse variance weighted | 26 | 0.047357 | 0.012937 | 0.000252 | 1.048496 | 1.022245 | 1.075422 | 0.035258 | Jingning et al. 2022 |  |
| VPS26A | psoriasis | Inverse variance weighted | 10 | 0.125637 | 0.026651 | 2.43E-06 | 1.13387 | 1.076162 | 1.194673 | 0.001247 | Jingning et al. 2022 |  |
| CXCL16 | psoriasis | Inverse variance weighted | 5 | -0.20218 | 0.047103 | 1.77E-05 | 0.816948 | 0.744903 | 0.895962 | 0.004545 | Jingning et al. 2022 |  |
| IL17RD | psoriasis | Inverse variance weighted | 14 | -0.1065 | 0.026627 | 6.34E-05 | 0.898974 | 0.853261 | 0.947135 | 0.01222 | Jingning et al. 2022 |  |
| TNXB | psoriasis | Inverse variance weighted | 15 | 0.445258 | 0.100994 | 1.04E-05 | 1.560893 | 1.280576 | 1.90257 | 0.003285 | Jingning et al. 2022 |  |
| ABO | psoriasis | Inverse variance weighted | 21 | 0.052058 | 0.012432 | 2.82E-05 | 1.053437 | 1.028079 | 1.07942 | 0.006212 | Jingning et al. 2022 |  |
| MAPRE1 | psoriasis | Wald ratio | 1 | -1.0583 | 0.269058 | 8.38E-05 | 0.347047 | 0.204815 | 0.588051 | 0.01597 | Ferkingsatad et al.2021 |  |
| BRD2 | psoriasis | Wald ratio | 1 | -1.44969 | 0.328542 | 1.02E-05 | 0.234643 | 0.123238 | 0.446753 | 0.002597 | Ferkingsatad et al.2021 |  |
| NEU1 | psoriasis | Wald ratio | 1 | -1.89837 | 0.328494 | 7.51E-09 | 0.149813 | 0.078692 | 0.285213 | 3.82E-06 | Ferkingsatad et al.2021 |  |
| PRSS8 | psoriasis | Wald ratio | 1 | 1.341709 | 0.259631 | 2.37E-07 | 3.825574 | 2.29982 | 6.363549 | 9.03E-05 | Ferkingsatad et al.2021 |  |
| AIF1 | psoriasis | Wald ratio | 1 | 0.552716 | 0.120607 | 4.59E-06 | 1.737966 | 1.372077 | 2.201426 | 0.001399 | Ferkingsatad et al.2021 |  |
| HSPA1A | psoriasis | Wald ratio | 1 | -1.5493 | 0.356137 | 1.36E-05 | 0.212397 | 0.105682 | 0.426873 | 0.002962 | Ferkingsatad et al.2021 |  |
| IER3 | psoriasis | Wald ratio | 1 | 6.027933 | 0.281192 | 6.03E-102 | 414.8566 | 239.0797 | 719.8687 | 9.19E-99 | Ferkingsatad et al.2021 |  |
| APOF | psoriasis | Inverse variance weighted | 3 | 0.609139 | 0.092481 | 4.50E-11 | 1.838847 | 1.533996 | 2.20428 | 3.43E-08 | Ferkingsatad et al.2021 |  |
| APOF | psoriasis | Wald ratio | 1 | 0.458963 | 0.098542 | 3.20E-06 | 1.582433 | 1.304501 | 1.919579 | 0.000592 | Sun_et al.2018 |  |
| HLA-DQA2 | psoriasis | Wald ratio | 1 | 0.217914 | 0.035428 | 7.70E-10 | 1.243481 | 1.160065 | 1.332895 | 4.27E-07 | Sun_et al.2018 |  |
| IL12BIL23A | psoriasis | Wald ratio | 1 | -0.3359 | 0.057859 | 6.42E-09 | 0.714691 | 0.638069 | 0.800514 | 1.78E-06 | Sun_et al.2018 |  |
| B3GNT2 | psoriasis | Wald ratio | 1 | -0.38283 | 0.095408 | 6.01E-05 | 0.681931 | 0.565623 | 0.822155 | 0.008336 | Sun_et al.2018 |  |
| nsnp, number of single nucleotide polymorphisms; b, beta; se, standard error; OR, odds ratio; CI, confidence interval; fdr, false discovery rate | | | | | | | | | | | | |

| Supplementary Table 7. Colocalization results | | |
| --- | --- | --- |
| GENE | corhort | PPH4 |
| AIF1 | Ferkingsatad et al.2021 | 1 |
| FCGR3A | Jingning et al. 2022 | 1 |
| NEU1 | Ferkingsatad et al.2021 | 1 |
| HSPA1A | Ferkingsatad et al.2021 | 0.9999997 |
| HSPA1A | Jingning et al. 2022 | 0.9999979 |
| TNXB | Jingning et al. 2022 | 0.9999979 |
| ABO | Jingning et al. 2022 | 0.9581235 |
| IER3 | Ferkingsatad et al.2021 | 0.7817984 |
| SWAP70 | Jingning et al. 2022 | 0.5613882 |
| B3GNT2 | Sun_et al.2018 | 0.5330456 |
| HLA-DQA2 | Sun_et al.2018 | 0.4754955 |
| IL17RD | Jingning et al. 2022 | 0.4643559 |
| PRSS8 | Jingning et al. 2022 | 0.4635096 |
| IL12B\|IL23A | Sun_et al.2018 | 0.3806027 |
| CXCL16 | Jingning et al. 2022 | 0.2817611 |
| IL7R | Jingning et al. 2022 | 0.281318 |
| APOF | Ferkingsatad et al.2021 | 0.21846371 |
| MAPRE1 | Jingning et al. 2022 | 0.1850339 |
| APOF | Sun_et al.2018 | 0.043456212 |
| VPS26A | Jingning et al. 2022 | 0.0317529 |

| Supplementary Table 8. Druggability results in the drugbank | | | | |
| --- | --- | --- | --- | --- |
| Protain | Gene | Drug Bank | | |
|  |  | General Function | Drug Name | Molecular Action |
| Fc gamma receptor IIIa | FCGR3A | Receptor for the Fc region of IgG | Cetuximab | binder |
|  |  |  | Etanercept | ligand |
|  |  |  | Human immunoglobulin G | antagonist |
|  |  |  | Alemtuzumab | binder |
|  |  |  | Gemtuzumab ozogamicin |  |
|  |  |  | Alefacept |  |
|  |  |  | Palivizumab |  |
|  |  |  | Daclizumab |  |
|  |  |  | Sarilumab |  |
|  |  |  | Benralizumab | binding |
|  |  |  | Catumaxomab | agonist |
|  |  |  | Amivantamab | inducer |
|  |  |  |  |  |
| neuraminidase 1 | NEU1 | Exo-alpha-sialidase activity | Oseltamivir | inhibitor |
|  |  |  | Acetylsalicylic acid | inhibitor |
|  |  |  | Celecoxib | inhibitor |

| Supplementary Table 9. PPI network results between psoriasis-related proteins and mature target proteins | | | | | | | | |
| --- | --- | --- | --- | --- | --- | --- | --- | --- |
| node1 | node2 | node1_string_id | node2_string_id | coexpression | experimentally_determined_interaction | database_annotated | automated_textmining | combined_score |
| AIF1 | FCGR3A | 9606.ENSP00000365227 | 9606.ENSP00000356946 | 0.367 | 0 | 0 | 0.571 | 0.717 |
| AIF1 | TRPV1 | 9606.ENSP00000365227 | 9606.ENSP00000459962 | 0.067 | 0.126 | 0 | 0.377 | 0.448 |
| AIF1 | IL17A | 9606.ENSP00000365227 | 9606.ENSP00000497968 | 0.059 | 0 | 0 | 0.445 | 0.455 |
| AIF1 | TNF | 9606.ENSP00000365227 | 9606.ENSP00000398698 | 0.156 | 0 | 0 | 0.826 | 0.846 |
| ATP4A | HMGCR | 9606.ENSP00000262623 | 9606.ENSP00000287936 | 0 | 0.053 | 0 | 0.507 | 0.514 |
| ATP4A | TNF | 9606.ENSP00000262623 | 9606.ENSP00000398698 | 0 | 0.071 | 0 | 0.54 | 0.554 |
| CD2 | RARA | 9606.ENSP00000358490 | 9606.ENSP00000254066 | 0.063 | 0 | 0 | 0.399 | 0.412 |
| CD2 | ITGAL | 9606.ENSP00000358490 | 9606.ENSP00000349252 | 0.354 | 0 | 0 | 0.89 | 0.925 |
| CD2 | FCGR3A | 9606.ENSP00000358490 | 9606.ENSP00000356946 | 0.263 | 0 | 0 | 0.899 | 0.922 |
| CD2 | JAK3 | 9606.ENSP00000358490 | 9606.ENSP00000432511 | 0.17 | 0.062 | 0 | 0.307 | 0.414 |
| CD2 | IL17A | 9606.ENSP00000358490 | 9606.ENSP00000497968 | 0.044 | 0 | 0 | 0.433 | 0.434 |
| CD2 | TNF | 9606.ENSP00000358490 | 9606.ENSP00000398698 | 0.129 | 0 | 0 | 0.54 | 0.582 |
| CYP27B1 | TNF | 9606.ENSP00000228606 | 9606.ENSP00000398698 | 0.056 | 0 | 0 | 0.422 | 0.431 |
| CYP27B1 | NR3C1 | 9606.ENSP00000228606 | 9606.ENSP00000231509 | 0 | 0.056 | 0 | 0.447 | 0.456 |
| CYP27B1 | RXRG | 9606.ENSP00000228606 | 9606.ENSP00000352900 | 0 | 0.788 | 0 | 0.213 | 0.826 |
| CYP27B1 | RXRA | 9606.ENSP00000228606 | 9606.ENSP00000419692 | 0 | 0.968 | 0 | 0.448 | 0.981 |
| DHFR | NR3C1 | 9606.ENSP00000396308 | 9606.ENSP00000231509 | 0.042 | 0 | 0 | 0.524 | 0.524 |
| DHFR | HMGCR | 9606.ENSP00000396308 | 9606.ENSP00000287936 | 0.06 | 0 | 0 | 0.37 | 0.432 |
| DHFR | FKBP1A | 9606.ENSP00000396308 | 9606.ENSP00000383003 | 0.06 | 0.292 | 0 | 0.516 | 0.65 |
| FCGR3A | IL17F | 9606.ENSP00000356946 | 9606.ENSP00000337432 | 0.086 | 0 | 0 | 0.376 | 0.405 |
| FCGR3A | ITGAL | 9606.ENSP00000356946 | 9606.ENSP00000349252 | 0.29 | 0 | 0 | 0.768 | 0.828 |
| FCGR3A | JAK2 | 9606.ENSP00000356946 | 9606.ENSP00000371067 | 0.118 | 0 | 0 | 0.37 | 0.42 |
| FCGR3A | IL17A | 9606.ENSP00000356946 | 9606.ENSP00000497968 | 0.063 | 0 | 0 | 0.804 | 0.808 |
| FCGR3A | TNF | 9606.ENSP00000356946 | 9606.ENSP00000398698 | 0.145 | 0 | 0 | 0.856 | 0.872 |
| FKBP1A | PDE4B | 9606.ENSP00000383003 | 9606.ENSP00000342637 | 0.047 | 0 | 0.54 | 0.056 | 0.55 |
| FKBP1A | PDE4D | 9606.ENSP00000383003 | 9606.ENSP00000345502 | 0.043 | 0 | 0.54 | 0.172 | 0.603 |
| FKBP1A | PPP3CA | 9606.ENSP00000383003 | 9606.ENSP00000378323 | 0.042 | 0.481 | 0.5 | 0.778 | 0.937 |
| FKBP1A | PPIA | 9606.ENSP00000383003 | 9606.ENSP00000419425 | 0.236 | 0.095 | 0 | 0.609 | 0.706 |
| HMGCR | RXRA | 9606.ENSP00000287936 | 9606.ENSP00000419692 | 0.044 | 0 | 0.4 | 0.294 | 0.559 |
| HMGCR | TNF | 9606.ENSP00000287936 | 9606.ENSP00000398698 | 0 | 0 | 0 | 0.572 | 0.572 |
| HSPA1A | NR3C1 | 9606.ENSP00000364802 | 9606.ENSP00000231509 | 0 | 0.354 | 0.5 | 0.278 | 0.746 |
| HSPA1A | TNF | 9606.ENSP00000364802 | 9606.ENSP00000398698 | 0.061 | 0 | 0 | 0.69 | 0.696 |
| IL12B | IL23A | 9606.ENSP00000231228 | 9606.ENSP00000228534 | 0.132 | 0.981 | 0.9 | 0.649 | 0.999 |
| IL12B | NR3C1 | 9606.ENSP00000231228 | 9606.ENSP00000231509 | 0.055 | 0.292 | 0 | 0.064 | 0.468 |
| IL12B | JAK1 | 9606.ENSP00000231228 | 9606.ENSP00000499900 | 0.055 | 0 | 0.5 | 0.134 | 0.555 |
| IL12B | JAK2 | 9606.ENSP00000231228 | 9606.ENSP00000371067 | 0.088 | 0 | 0.54 | 0.209 | 0.639 |
| IL12B | TNF | 9606.ENSP00000231228 | 9606.ENSP00000398698 | 0.17 | 0 | 0.4 | 0.472 | 0.714 |
| IL12B | TYK2 | 9606.ENSP00000231228 | 9606.ENSP00000431885 | 0.055 | 0 | 0.54 | 0.513 | 0.769 |
| IL17A | IL23A | 9606.ENSP00000497968 | 9606.ENSP00000228534 | 0.089 | 0 | 0.4 | 0.867 | 0.92 |
| IL17A | IL1RL2 | 9606.ENSP00000497968 | 9606.ENSP00000264257 | 0.115 | 0 | 0 | 0.529 | 0.565 |
| IL17A | IL1RAP | 9606.ENSP00000497968 | 9606.ENSP00000314807 | 0 | 0 | 0 | 0.4 | 0.4 |
| IL17A | IL17RA | 9606.ENSP00000497968 | 9606.ENSP00000320936 | 0 | 0.962 | 0.9 | 0.999 | 0.999 |
| IL17A | IL17F | 9606.ENSP00000497968 | 9606.ENSP00000337432 | 0.339 | 0.9 | 0.9 | 0.203 | 0.994 |
| IL17A | ITGAL | 9606.ENSP00000497968 | 9606.ENSP00000349252 | 0 | 0 | 0 | 0.473 | 0.473 |
| IL17A | PDE4A | 9606.ENSP00000497968 | 9606.ENSP00000370078 | 0 | 0 | 0 | 0.479 | 0.479 |
| IL17A | JAK2 | 9606.ENSP00000497968 | 9606.ENSP00000371067 | 0 | 0 | 0 | 0.599 | 0.599 |
| IL17A | TNF | 9606.ENSP00000497968 | 9606.ENSP00000398698 | 0.068 | 0 | 0 | 0.957 | 0.958 |
| IL17A | TYK2 | 9606.ENSP00000497968 | 9606.ENSP00000431885 | 0 | 0 | 0 | 0.583 | 0.583 |
| IL17A | JAK3 | 9606.ENSP00000497968 | 9606.ENSP00000432511 | 0.044 | 0 | 0 | 0.528 | 0.529 |
| IL17A | JAK1 | 9606.ENSP00000497968 | 9606.ENSP00000499900 | 0 | 0 | 0 | 0.598 | 0.598 |
| IL17F | IL23A | 9606.ENSP00000337432 | 9606.ENSP00000228534 | 0.078 | 0 | 0.4 | 0.869 | 0.921 |
| IL17F | IL17RA | 9606.ENSP00000337432 | 9606.ENSP00000320936 | 0 | 0.905 | 0.9 | 0.999 | 0.999 |
| IL17F | JAK2 | 9606.ENSP00000337432 | 9606.ENSP00000371067 | 0.083 | 0 | 0 | 0.395 | 0.422 |
| IL17F | TYK2 | 9606.ENSP00000337432 | 9606.ENSP00000431885 | 0.099 | 0 | 0 | 0.471 | 0.503 |
| IL17F | TNF | 9606.ENSP00000337432 | 9606.ENSP00000398698 | 0.098 | 0 | 0 | 0.821 | 0.832 |
| IL17RA | IL23A | 9606.ENSP00000320936 | 9606.ENSP00000228534 | 0 | 0 | 0 | 0.668 | 0.668 |
| IL17RA | JAK2 | 9606.ENSP00000320936 | 9606.ENSP00000371067 | 0.122 | 0.066 | 0 | 0.359 | 0.429 |
| IL17RA | JAK1 | 9606.ENSP00000320936 | 9606.ENSP00000499900 | 0.117 | 0.066 | 0 | 0.573 | 0.617 |
| IL17RA | TYK2 | 9606.ENSP00000320936 | 9606.ENSP00000431885 | 0.09 | 0.151 | 0 | 0.649 | 0.706 |
| IL17RA | TNF | 9606.ENSP00000320936 | 9606.ENSP00000398698 | 0.128 | 0 | 0 | 0.808 | 0.825 |
| IL1RAP | IL1RL2 | 9606.ENSP00000314807 | 9606.ENSP00000264257 | 0.074 | 0.328 | 0.5 | 0.987 | 0.995 |
| IL1RAP | TNF | 9606.ENSP00000314807 | 9606.ENSP00000398698 | 0.054 | 0 | 0 | 0.447 | 0.454 |
| IL1RL2 | TNF | 9606.ENSP00000264257 | 9606.ENSP00000398698 | 0 | 0 | 0 | 0.418 | 0.418 |
| IL23A | TNF | 9606.ENSP00000228534 | 9606.ENSP00000398698 | 0.144 | 0 | 0 | 0.624 | 0.664 |
| IL23A | JAK2 | 9606.ENSP00000228534 | 9606.ENSP00000371067 | 0 | 0 | 0.9 | 0.368 | 0.934 |
| IL23A | TYK2 | 9606.ENSP00000228534 | 9606.ENSP00000431885 | 0 | 0 | 0.9 | 0.399 | 0.937 |
| ITGAL | TNF | 9606.ENSP00000349252 | 9606.ENSP00000398698 | 0.153 | 0 | 0 | 0.605 | 0.71 |
| JAK1 | JAK2 | 9606.ENSP00000499900 | 9606.ENSP00000371067 | 0.113 | 0.51 | 0.9 | 0.982 | 0.999 |
| JAK1 | TNF | 9606.ENSP00000499900 | 9606.ENSP00000398698 | 0.096 | 0.49 | 0 | 0.658 | 0.828 |
| JAK1 | TYK2 | 9606.ENSP00000499900 | 9606.ENSP00000431885 | 0.049 | 0.292 | 0.9 | 0.983 | 0.998 |
| JAK1 | JAK3 | 9606.ENSP00000499900 | 9606.ENSP00000432511 | 0.057 | 0.51 | 0.9 | 0.983 | 0.999 |
| JAK2 | RXRA | 9606.ENSP00000371067 | 9606.ENSP00000419692 | 0.107 | 0.071 | 0 | 0.371 | 0.433 |
| JAK2 | PPIA | 9606.ENSP00000371067 | 9606.ENSP00000419425 | 0.068 | 0.348 | 0 | 0.178 | 0.457 |
| JAK2 | TNF | 9606.ENSP00000371067 | 9606.ENSP00000398698 | 0.118 | 0.057 | 0 | 0.78 | 0.801 |
| JAK2 | JAK3 | 9606.ENSP00000371067 | 9606.ENSP00000432511 | 0.045 | 0.292 | 0.9 | 0.681 | 0.975 |
| JAK2 | TYK2 | 9606.ENSP00000371067 | 9606.ENSP00000431885 | 0.089 | 0.292 | 0.8 | 0.982 | 0.997 |
| JAK3 | TNF | 9606.ENSP00000432511 | 9606.ENSP00000398698 | 0.118 | 0.057 | 0 | 0.517 | 0.563 |
| JAK3 | TYK2 | 9606.ENSP00000432511 | 9606.ENSP00000431885 | 0.085 | 0.292 | 0 | 0.864 | 0.906 |
| KEAP1 | TNF | 9606.ENSP00000377245 | 9606.ENSP00000398698 | 0 | 0 | 0 | 0.591 | 0.591 |
| NR3C1 | TRPV1 | 9606.ENSP00000231509 | 9606.ENSP00000459962 | 0 | 0 | 0 | 0.461 | 0.461 |
| NR3C1 | RARA | 9606.ENSP00000231509 | 9606.ENSP00000254066 | 0 | 0.07 | 0 | 0.507 | 0.521 |
| NR3C1 | RXRA | 9606.ENSP00000231509 | 9606.ENSP00000419692 | 0.067 | 0 | 0 | 0.525 | 0.538 |
| NR3C1 | RXRB | 9606.ENSP00000231509 | 9606.ENSP00000363817 | 0.055 | 0.292 | 0 | 0.47 | 0.614 |
| NR3C1 | TNF | 9606.ENSP00000231509 | 9606.ENSP00000398698 | 0.086 | 0 | 0 | 0.733 | 0.745 |
| PDE4A | PDE4D | 9606.ENSP00000370078 | 9606.ENSP00000345502 | 0.045 | 0.238 | 0.4 | 0.347 | 0.682 |
| PDE4A | TNF | 9606.ENSP00000370078 | 9606.ENSP00000398698 | 0 | 0 | 0 | 0.576 | 0.576 |
| PDE4B | TNF | 9606.ENSP00000342637 | 9606.ENSP00000398698 | 0.058 | 0 | 0 | 0.39 | 0.401 |
| PDE4B | PDE4D | 9606.ENSP00000342637 | 9606.ENSP00000345502 | 0.122 | 0.422 | 0.54 | 0.069 | 0.757 |
| PPIA | PPP3CA | 9606.ENSP00000419425 | 9606.ENSP00000378323 | 0.053 | 0.946 | 0.4 | 0.584 | 0.985 |
| PPIA | TNF | 9606.ENSP00000419425 | 9606.ENSP00000398698 | 0.056 | 0 | 0 | 0.449 | 0.457 |
| RARA | RARG | 9606.ENSP00000254066 | 9606.ENSP00000388510 | 0.116 | 0.78 | 0.5 | 0.62 | 0.958 |
| RARA | RXRG | 9606.ENSP00000254066 | 9606.ENSP00000352900 | 0.068 | 0.848 | 0.8 | 0.476 | 0.983 |
| RARA | RARB | 9606.ENSP00000254066 | 9606.ENSP00000332296 | 0 | 0.921 | 0.5 | 0.638 | 0.984 |
| RARA | RXRB | 9606.ENSP00000254066 | 9606.ENSP00000363817 | 0.104 | 0.862 | 0.8 | 0.756 | 0.993 |
| RARA | RXRA | 9606.ENSP00000254066 | 9606.ENSP00000419692 | 0.113 | 0.991 | 0.9 | 0.983 | 0.999 |
| RARB | RARG | 9606.ENSP00000332296 | 9606.ENSP00000388510 | 0.06 | 0 | 0.5 | 0.824 | 0.911 |
| RARB | RXRG | 9606.ENSP00000332296 | 9606.ENSP00000352900 | 0.111 | 0.642 | 0.8 | 0.63 | 0.973 |
| RARB | RXRB | 9606.ENSP00000332296 | 9606.ENSP00000363817 | 0.067 | 0.716 | 0.9 | 0.754 | 0.992 |
| RARB | RXRA | 9606.ENSP00000332296 | 9606.ENSP00000419692 | 0.068 | 0.971 | 0.8 | 0.47 | 0.996 |
| RARG | RXRG | 9606.ENSP00000388510 | 9606.ENSP00000352900 | 0.068 | 0.345 | 0.9 | 0.408 | 0.959 |
| RARG | RXRB | 9606.ENSP00000388510 | 9606.ENSP00000363817 | 0.081 | 0.786 | 0.5 | 0.52 | 0.946 |
| RARG | RXRA | 9606.ENSP00000388510 | 9606.ENSP00000419692 | 0.088 | 0.208 | 0.7 | 0.777 | 0.945 |
| RXRA | RXRG | 9606.ENSP00000419692 | 9606.ENSP00000352900 | 0 | 0.432 | 0.9 | 0.064 | 0.943 |
| RXRA | RXRB | 9606.ENSP00000419692 | 9606.ENSP00000363817 | 0.06 | 0 | 0.9 | 0.347 | 0.934 |
| RXRB | RXRG | 9606.ENSP00000363817 | 9606.ENSP00000352900 | 0 | 0 | 0.9 | 0.4 | 0.938 |
| TNF | TRPV1 | 9606.ENSP00000398698 | 9606.ENSP00000459962 | 0 | 0 | 0 | 0.592 | 0.592 |
| TNF | TYK2 | 9606.ENSP00000398698 | 9606.ENSP00000431885 | 0.092 | 0.057 | 0 | 0.718 | 0.737 |

| Supplementary Table 10. Effects after knocking out psoriasis-relative genes | | | | | | | |
| --- | --- | --- | --- | --- | --- | --- | --- |
| Gene | Allelic Composition | Genetic Background | Genotype ID | Qualifier | Annotated Term | Phenotype Summary Category | Reference |
| AIF1 | Aif1<tm1.1(KOMP)Wtsi>/Aif1<tm1.1(KOMP)Wtsi> | C57BL/6N-Aif1<tm1.1(KOMP)Wtsi>/Ieg | MGI:5797349 |  | abnormal lens morphology | vision/eye | J:211773 |
| AIF1 | Aif1<tm1.1(KOMP)Wtsi>/Aif1<tm1.1(KOMP)Wtsi> | C57BL/6N-Aif1<tm1.1(KOMP)Wtsi>/Ieg | MGI:5797349 |  | abnormal optic disk morphology | nervous system | J:211773 |
| AIF1 | Aif1<tm1.1(KOMP)Wtsi>/Aif1<tm1.1(KOMP)Wtsi> | C57BL/6N-Aif1<tm1.1(KOMP)Wtsi>/Ieg | MGI:5797349 |  | abnormal optic disk morphology | vision/eye | J:211773 |
| AIF1 | Aif1<tm1.1(KOMP)Wtsi>/Aif1<tm1.1(KOMP)Wtsi> | C57BL/6N-Aif1<tm1.1(KOMP)Wtsi>/Ieg | MGI:5797349 |  | abnormal retina blood vessel morphology | cardiovascular system | J:211773 |
| AIF1 | Aif1<tm1.1(KOMP)Wtsi>/Aif1<tm1.1(KOMP)Wtsi> | C57BL/6N-Aif1<tm1.1(KOMP)Wtsi>/Ieg | MGI:5797349 |  | abnormal retina blood vessel morphology | vision/eye | J:211773 |
| AIF1 | Aif1<tm1.1(KOMP)Wtsi>/Aif1<tm1.1(KOMP)Wtsi> | C57BL/6N-Aif1<tm1.1(KOMP)Wtsi>/Ieg | MGI:5797349 |  | abnormal retina morphology | vision/eye | J:211773 |
| AIF1 | Aif1<tm1.1(KOMP)Wtsi>/Aif1<tm1.1(KOMP)Wtsi> | C57BL/6N-Aif1<tm1.1(KOMP)Wtsi>/Ieg | MGI:5797349 |  | abnormal vitreous body morphology | vision/eye | J:211773 |
| AIF1 | Aif1<tm1.1(KOMP)Wtsi>/Aif1<tm1.1(KOMP)Wtsi> | C57BL/6N-Aif1<tm1.1(KOMP)Wtsi>/Ieg | MGI:5797349 |  | decreased blood urea nitrogen level | homeostasis/metabolism | J:211773 |
| AIF1 | Aif1<tm1.1(KOMP)Wtsi>/Aif1<tm1.1(KOMP)Wtsi> | C57BL/6N-Aif1<tm1.1(KOMP)Wtsi>/Ieg | MGI:5797349 |  | decreased lean body mass | growth/size/body region | J:211773 |
| AIF1 | Aif1<tm1Nsib>/Aif1<tm1Nsib> | involves: 129S/SvEv * C57BL/6 | MGI:5547994 |  | decreased spleen weight | hematopoietic system | J:204462 |
| AIF1 | Aif1<tm1Nsib>/Aif1<tm1Nsib> | involves: 129S/SvEv * C57BL/6 | MGI:5547994 |  | decreased spleen weight | immune system | J:204462 |
| AIF1 | Aif1<tm1Nsib>/Aif1<tm1Nsib> | involves: 129S/SvEv * C57BL/6 | MGI:5547994 |  | decreased susceptibility to induced arthritis | immune system | J:204462 |
| AIF1 | Aif1<tm1Nsib>/Aif1<tm1Nsib> | involves: 129S/SvEv * C57BL/6 | MGI:5547994 |  | decreased susceptibility to induced arthritis | skeleton | J:204462 |
| AIF1 | Aif1<tm1Nsib>/Aif1<tm1Nsib> | involves: 129S/SvEv * C57BL/6 | MGI:5547994 |  | thrombocytopenia | hematopoietic system | J:204462 |
| FCGR3A | Fcgr3<tm1b(EUCOMM)Hmgu>/Fcgr3<tm1b(EUCOMM)Hmgu> | C57BL/6N-Fcgr3<tm1b(EUCOMM)Hmgu>/H | MGI:6262430 |  | abnormal gait | behavior/neurological | J:211773 |
| FCGR3A | Fcgr3<tm1b(EUCOMM)Hmgu>/Fcgr3<tm1b(EUCOMM)Hmgu> | C57BL/6N-Fcgr3<tm1b(EUCOMM)Hmgu>/H | MGI:6262430 |  | decreased circulating calcium level | homeostasis/metabolism | J:211773 |
| FCGR3A | Fcgr3<tm1b(EUCOMM)Hmgu>/Fcgr3<tm1b(EUCOMM)Hmgu> | C57BL/6N-Fcgr3<tm1b(EUCOMM)Hmgu>/H | MGI:6262430 |  | decreased circulating creatine kinase level | homeostasis/metabolism | J:211773 |
| FCGR3A | Fcgr3<tm1b(EUCOMM)Hmgu>/Fcgr3<tm1b(EUCOMM)Hmgu> | C57BL/6N-Fcgr3<tm1b(EUCOMM)Hmgu>/H | MGI:6262430 |  | decreased circulating serum albumin level | homeostasis/metabolism | J:211773 |
| FCGR3A | Fcgr3<tm1b(EUCOMM)Hmgu>/Fcgr3<tm1b(EUCOMM)Hmgu> | C57BL/6N-Fcgr3<tm1b(EUCOMM)Hmgu>/H | MGI:6262430 |  | decreased circulating total protein level | homeostasis/metabolism | J:211773 |
| FCGR3A | Fcgr3<tm1b(EUCOMM)Hmgu>/Fcgr3<tm1b(EUCOMM)Hmgu> | C57BL/6N-Fcgr3<tm1b(EUCOMM)Hmgu>/H | MGI:6262430 |  | increased anxiety-related response | behavior/neurological | J:211773 |
| FCGR3A | Fcgr3<tm1b(EUCOMM)Hmgu>/Fcgr3<tm1b(EUCOMM)Hmgu> | C57BL/6N-Fcgr3<tm1b(EUCOMM)Hmgu>/H | MGI:6262430 |  | increased heart rate | cardiovascular system | J:211773 |
| FCGR3A | Fcgr3<tm1b(EUCOMM)Hmgu>/Fcgr3<tm1b(EUCOMM)Hmgu> | C57BL/6N-Fcgr3<tm1b(EUCOMM)Hmgu>/H | MGI:6262430 |  | tremors | behavior/neurological | J:211773 |
| FCGR3A | Fcgr3<tm1Jsv>/Fcgr3<tm1Jsv> | B6.129P2-Fcgr3<tm1Jsv>/J | MGI:3052641 |  | abnormal leukocyte cell number | hematopoietic system | J:78297 |
| FCGR3A | Fcgr3<tm1Jsv>/Fcgr3<tm1Jsv> | B6.129P2-Fcgr3<tm1Jsv>/J | MGI:3052641 |  | abnormal leukocyte cell number | immune system | J:78297 |
| FCGR3A | Fcgr3<tm1Jsv>/Fcgr3<tm1Jsv> | B6.129P2-Fcgr3<tm1Jsv>/J | MGI:3052641 |  | abnormal neutrophil physiology | hematopoietic system | J:78297 |
| FCGR3A | Fcgr3<tm1Jsv>/Fcgr3<tm1Jsv> | B6.129P2-Fcgr3<tm1Jsv>/J | MGI:3052641 |  | abnormal neutrophil physiology | immune system | J:78297 |
| FCGR3A | Fcgr3<tm1Jsv>/Fcgr3<tm1Jsv> | B6.129P2-Fcgr3<tm1Jsv>/J | MGI:3052641 |  | decreased susceptibility to experimental autoimmune encephalomyelitis | immune system | J:126520 |
| FCGR3A | Fcgr3<tm1Jsv>/Fcgr3<tm1Jsv> | B6.129P2-Fcgr3<tm1Jsv>/J | MGI:3052641 |  | decreased susceptibility to induced arthritis | immune system | J:106191 |
| FCGR3A | Fcgr3<tm1Jsv>/Fcgr3<tm1Jsv> | B6.129P2-Fcgr3<tm1Jsv>/J | MGI:3052641 |  | decreased susceptibility to induced arthritis | skeleton | J:106191 |
| FCGR3A | Fcgr3<tm1Jsv>/Fcgr3<tm1Jsv> | B6.129P2-Fcgr3<tm1Jsv>/J | MGI:3052641 |  | impaired neutrophil recruitment | hematopoietic system | J:78297 |
| FCGR3A | Fcgr3<tm1Jsv>/Fcgr3<tm1Jsv> | B6.129P2-Fcgr3<tm1Jsv>/J | MGI:3052641 |  | impaired neutrophil recruitment | immune system | J:78297 |
| FCGR3A | Fcgr3<tm1Jsv>/Fcgr3<tm1Jsv> | B6.129P2-Fcgr3<tm1Jsv>/J | MGI:3052641 |  | increased neutrophil cell number | hematopoietic system | J:78297 |
| FCGR3A | Fcgr3<tm1Jsv>/Fcgr3<tm1Jsv> | B6.129P2-Fcgr3<tm1Jsv>/J | MGI:3052641 |  | increased neutrophil cell number | immune system | J:78297 |
| FCGR3A | Fcgr3<tm1Jsv>/Fcgr3<tm1Jsv> | involves: 129P2/OlaHsd * C57BL/6 | MGI:2664960 |  | abnormal dendritic cell antigen presentation | immune system | J:83995 |
| FCGR3A | Fcgr3<tm1Jsv>/Fcgr3<tm1Jsv> | involves: 129P2/OlaHsd * C57BL/6 | MGI:2664960 |  | abnormal type I hypersensitivity reaction | immune system | J:83995 |
| FCGR3A | Fcgr3<tm1Jsv>/Fcgr3<tm1Jsv> | involves: 129P2/OlaHsd * C57BL/6 | MGI:2664960 |  | decreased mast cell degranulation | cellular | J:35057 |
| FCGR3A | Fcgr3<tm1Jsv>/Fcgr3<tm1Jsv> | involves: 129P2/OlaHsd * C57BL/6 | MGI:2664960 |  | decreased mast cell degranulation | hematopoietic system | J:35057 |
| FCGR3A | Fcgr3<tm1Jsv>/Fcgr3<tm1Jsv> | involves: 129P2/OlaHsd * C57BL/6 | MGI:2664960 |  | decreased mast cell degranulation | immune system | J:35057 |
| FCGR3A | Fcgr3<tm1Jsv>/Fcgr3<tm1Jsv> | involves: 129P2/OlaHsd * C57BL/6 | MGI:2664960 |  | decreased susceptibility to autoimmune hemolytic anemia | hematopoietic system | J:83995 |
| FCGR3A | Fcgr3<tm1Jsv>/Fcgr3<tm1Jsv> | involves: 129P2/OlaHsd * C57BL/6 | MGI:2664960 |  | decreased susceptibility to autoimmune hemolytic anemia | immune system | J:83995 |
| FCGR3A | Fcgr3<tm1Jsv>/Fcgr3<tm1Jsv> | involves: 129P2/OlaHsd * C57BL/6 | MGI:2664960 |  | decreased susceptibility to type II hypersensitivity reaction | immune system | J:35057 |
| FCGR3A | Fcgr3<tm1Jsv>/Fcgr3<tm1Jsv> | involves: 129P2/OlaHsd * C57BL/6 | MGI:2664960 |  | impaired macrophage phagocytosis | cellular | J:35057 |
| FCGR3A | Fcgr3<tm1Jsv>/Fcgr3<tm1Jsv> | involves: 129P2/OlaHsd * C57BL/6 | MGI:2664960 |  | impaired macrophage phagocytosis | hematopoietic system | J:35057 |
| FCGR3A | Fcgr3<tm1Jsv>/Fcgr3<tm1Jsv> | involves: 129P2/OlaHsd * C57BL/6 | MGI:2664960 |  | impaired macrophage phagocytosis | immune system | J:35057 |
| FCGR3A | Fcgr3<tm1Jsv>/Fcgr3<tm1Jsv> | involves: 129P2/OlaHsd * C57BL/6 | MGI:2664960 |  | impaired natural killer cell mediated cytotoxicity | hematopoietic system | J:35057 |
| FCGR3A | Fcgr3<tm1Jsv>/Fcgr3<tm1Jsv> | involves: 129P2/OlaHsd * C57BL/6 | MGI:2664960 |  | impaired natural killer cell mediated cytotoxicity | immune system | J:35057 |
| FCGR3A | Fcgr3<tm1Ttk>/Fcgr3<tm1Ttk> | involves: 129X1/SvJ | MGI:3623226 |  | decreased susceptibility to type I hypersensitivity reaction | immune system | J:107371 |
| FCGR3A | Fcer1a<tm1Knt>/Fcer1a<tm1Knt> Fcer2a<tm1Max>/Fcer2a<tm1Max> Fcgr1<tm1Jsv>/Fcgr1<tm1Jsv> Fcgr2b<tm1Ttk>/Fcgr2b<tm1Ttk> Fcgr3<tm1Jsv>/Fcgr3<tm1Jsv> | B6.Cg-Fcgr2b<tm1Ttk> Fcgr3<tm1Jsv> Fcer1a<tm1Knt> Fcgr1<tm1Jsv> Fcer2a<tm1Max> | MGI:3851204 |  | abnormal macrophage physiology | hematopoietic system | J:144620 |
| FCGR3A | Fcer1a<tm1Knt>/Fcer1a<tm1Knt> Fcer2a<tm1Max>/Fcer2a<tm1Max> Fcgr1<tm1Jsv>/Fcgr1<tm1Jsv> Fcgr2b<tm1Ttk>/Fcgr2b<tm1Ttk> Fcgr3<tm1Jsv>/Fcgr3<tm1Jsv> | B6.Cg-Fcgr2b<tm1Ttk> Fcgr3<tm1Jsv> Fcer1a<tm1Knt> Fcgr1<tm1Jsv> Fcer2a<tm1Max> | MGI:3851204 |  | abnormal macrophage physiology | immune system | J:144620 |
| FCGR3A | Fcer1a<tm1Knt>/Fcer1a<tm1Knt> Fcer2a<tm1Max>/Fcer2a<tm1Max> Fcgr1<tm1Jsv>/Fcgr1<tm1Jsv> Fcgr2b<tm1Ttk>/Fcgr2b<tm1Ttk> Fcgr3<tm1Jsv>/Fcgr3<tm1Jsv> | B6.Cg-Fcgr2b<tm1Ttk> Fcgr3<tm1Jsv> Fcer1a<tm1Knt> Fcgr1<tm1Jsv> Fcer2a<tm1Max> | MGI:3851204 |  | abnormal mast cell physiology | hematopoietic system | J:144620 |
| FCGR3A | Fcer1a<tm1Knt>/Fcer1a<tm1Knt> Fcer2a<tm1Max>/Fcer2a<tm1Max> Fcgr1<tm1Jsv>/Fcgr1<tm1Jsv> Fcgr2b<tm1Ttk>/Fcgr2b<tm1Ttk> Fcgr3<tm1Jsv>/Fcgr3<tm1Jsv> | B6.Cg-Fcgr2b<tm1Ttk> Fcgr3<tm1Jsv> Fcer1a<tm1Knt> Fcgr1<tm1Jsv> Fcer2a<tm1Max> | MGI:3851204 |  | abnormal mast cell physiology | immune system | J:144620 |
| FCGR3A | Fcer1a<tm1Knt>/Fcer1a<tm1Knt> Fcer2a<tm1Max>/Fcer2a<tm1Max> Fcgr1<tm1Jsv>/Fcgr1<tm1Jsv> Fcgr2b<tm1Ttk>/Fcgr2b<tm1Ttk> Fcgr3<tm1Jsv>/Fcgr3<tm1Jsv> | B6.Cg-Fcgr2b<tm1Ttk> Fcgr3<tm1Jsv> Fcer1a<tm1Knt> Fcgr1<tm1Jsv> Fcer2a<tm1Max> | MGI:3851204 |  | increased susceptibility to type I hypersensitivity reaction | immune system | J:144620 |
| FCGR3A | Fcgr1<tm1Jsv>/Fcgr1<tm1Jsv> Fcgr2b<tm1Ttk>/Fcgr2b<tm1Ttk> Fcgr3<tm1Jsv>/Fcgr3<tm1Jsv> | involves: 129P2/OlaHsd * 129S4/SvJae * BALB/c * C57BL/6 | MGI:2664970 |  | impaired macrophage phagocytosis | cellular | J:83995 |
| FCGR3A | Fcgr1<tm1Jsv>/Fcgr1<tm1Jsv> Fcgr2b<tm1Ttk>/Fcgr2b<tm1Ttk> Fcgr3<tm1Jsv>/Fcgr3<tm1Jsv> | involves: 129P2/OlaHsd * 129S4/SvJae * BALB/c * C57BL/6 | MGI:2664970 |  | impaired macrophage phagocytosis | hematopoietic system | J:83995 |
| FCGR3A | Fcgr1<tm1Jsv>/Fcgr1<tm1Jsv> Fcgr2b<tm1Ttk>/Fcgr2b<tm1Ttk> Fcgr3<tm1Jsv>/Fcgr3<tm1Jsv> | involves: 129P2/OlaHsd * 129S4/SvJae * BALB/c * C57BL/6 | MGI:2664970 |  | impaired macrophage phagocytosis | immune system | J:83995 |
| FCGR3A | Fcgr3<tm1Jsv>/Fcgr3<tm1Jsv> Sle21<129P2/Ola>/Sle21<129P2/Ola> | B6.129P2-Fcgr3<tm1Jsv> | MGI:5427952 |  | enlarged spleen | growth/size/body region | J:179175 |
| FCGR3A | Fcgr3<tm1Jsv>/Fcgr3<tm1Jsv> Sle21<129P2/Ola>/Sle21<129P2/Ola> | B6.129P2-Fcgr3<tm1Jsv> | MGI:5427952 |  | enlarged spleen | hematopoietic system | J:179175 |
| FCGR3A | Fcgr3<tm1Jsv>/Fcgr3<tm1Jsv> Sle21<129P2/Ola>/Sle21<129P2/Ola> | B6.129P2-Fcgr3<tm1Jsv> | MGI:5427952 |  | enlarged spleen | immune system | J:179175 |
| FCGR3A | Fcgr3<tm1Jsv>/Fcgr3<tm1Jsv> Sle21<129P2/Ola>/Sle21<129P2/Ola> | B6.129P2-Fcgr3<tm1Jsv> | MGI:5427952 |  | increased activated T cell number | hematopoietic system | J:179175 |
| FCGR3A | Fcgr3<tm1Jsv>/Fcgr3<tm1Jsv> Sle21<129P2/Ola>/Sle21<129P2/Ola> | B6.129P2-Fcgr3<tm1Jsv> | MGI:5427952 |  | increased activated T cell number | immune system | J:179175 |
| FCGR3A | Fcgr3<tm1Jsv>/Fcgr3<tm1Jsv> Sle21<129P2/Ola>/Sle21<129P2/Ola> | B6.129P2-Fcgr3<tm1Jsv> | MGI:5427952 |  | increased anti-chromatin antibody level | immune system | J:179175 |
| FCGR3A | Fcgr3<tm1Jsv>/Fcgr3<tm1Jsv> Sle21<129P2/Ola>/Sle21<129P2/Ola> | B6.129P2-Fcgr3<tm1Jsv> | MGI:5427952 |  | increased anti-double stranded DNA antibody level | immune system | J:179175 |
| FCGR3A | Fcgr3<tm1Jsv>/Fcgr3<tm1Jsv> Sle21<129P2/Ola>/Sle21<129P2/Ola> | B6.129P2-Fcgr3<tm1Jsv> | MGI:5427952 |  | increased anti-histone antibody level | immune system | J:179175 |
| FCGR3A | Fcgr3<tm1Jsv>/Fcgr3<tm1Jsv> Sle21<129P2/Ola>/Sle21<129P2/Ola> | B6.129P2-Fcgr3<tm1Jsv> | MGI:5427952 |  | increased anti-single stranded DNA antibody level | immune system | J:179175 |
| FCGR3A | Fcgr3<tm1Jsv>/Fcgr3<tm1Jsv> Sle21<129P2/Ola>/Sle21<129P2/Ola> | B6.129P2-Fcgr3<tm1Jsv> | MGI:5427952 |  | increased B cell number | hematopoietic system | J:179175 |
| FCGR3A | Fcgr3<tm1Jsv>/Fcgr3<tm1Jsv> Sle21<129P2/Ola>/Sle21<129P2/Ola> | B6.129P2-Fcgr3<tm1Jsv> | MGI:5427952 |  | increased B cell number | immune system | J:179175 |
| FCGR3A | Fcgr3<tm1Jsv>/Fcgr3<tm1Jsv> Sle21<129P2/Ola>/Sle21<129P2/Ola> | B6.129P2-Fcgr3<tm1Jsv> | MGI:5427952 |  | increased spleen weight | growth/size/body region | J:179175 |
| FCGR3A | Fcgr3<tm1Jsv>/Fcgr3<tm1Jsv> Sle21<129P2/Ola>/Sle21<129P2/Ola> | B6.129P2-Fcgr3<tm1Jsv> | MGI:5427952 |  | increased spleen weight | hematopoietic system | J:179175 |
| FCGR3A | Fcgr3<tm1Jsv>/Fcgr3<tm1Jsv> Sle21<129P2/Ola>/Sle21<129P2/Ola> | B6.129P2-Fcgr3<tm1Jsv> | MGI:5427952 |  | increased spleen weight | immune system | J:179175 |
| FCGR3A | Fcgr3<tm1Jsv>/Fcgr3<tm1Jsv> Sle21<129P2/Ola>/Sle21<129P2/Ola> | B6.129P2-Fcgr3<tm1Jsv> | MGI:5427952 | normal | mortality/aging | mortality/aging | J:179175 |
| NEU1 | Neu1<a>/Neu1<a> | B6.SM-Neu1<a> | MGI:3843003 |  | abnormal enzyme/coenzyme activity | homeostasis/metabolism | J:147881 |
| NEU1 | Neu1<a>/Neu1<a> | B6.SM-Neu1<a> | MGI:3843003 |  | decreased body weight | growth/size/body region | J:147881 |
| NEU1 | Neu1<a>/Neu1<a> | SM/J | MGI:3719077 |  | abnormal enzyme/coenzyme activity | homeostasis/metabolism | J:43930 |
| NEU1 | Neu1<a>/Neu1<a> | SM/J | MGI:3719077 |  | abnormal enzyme/coenzyme activity | homeostasis/metabolism | J:147881 |
| NEU1 | Neu1<a>/Neu1<a> | SM/J | MGI:3719077 |  | abnormal Kupffer cell morphology | cardiovascular system | J:147881 |
| NEU1 | Neu1<a>/Neu1<a> | SM/J | MGI:3719077 |  | abnormal Kupffer cell morphology | hematopoietic system | J:147881 |
| NEU1 | Neu1<a>/Neu1<a> | SM/J | MGI:3719077 |  | abnormal Kupffer cell morphology | immune system | J:147881 |
| NEU1 | Neu1<a>/Neu1<a> | SM/J | MGI:3719077 |  | abnormal Kupffer cell morphology | liver/biliary system | J:147881 |
| NEU1 | Neu1<a>/Neu1<a> | SM/J | MGI:3719077 |  | abnormal proximal convoluted tubule morphology | renal/urinary system | J:147881 |
| NEU1 | Neu1<a>/Neu1<a> | SM/J | MGI:3719077 |  | decreased body weight | growth/size/body region | J:147881 |
| NEU1 | Neu1<tm1Adz>/Neu1<tm1Adz> | either: (involves: 129S1/Sv * C57BL/6) or (involves: 129S1/Sv * NMRI) | MGI:3719098 |  | abnormal choroid plexus morphology | nervous system | J:76937 |
| NEU1 | Neu1<tm1Adz>/Neu1<tm1Adz> | either: (involves: 129S1/Sv * C57BL/6) or (involves: 129S1/Sv * NMRI) | MGI:3719098 |  | abnormal gait | behavior/neurological | J:76937 |
| NEU1 | Neu1<tm1Adz>/Neu1<tm1Adz> | either: (involves: 129S1/Sv * C57BL/6) or (involves: 129S1/Sv * NMRI) | MGI:3719098 |  | abnormal Kupffer cell morphology | cardiovascular system | J:76937 |
| NEU1 | Neu1<tm1Adz>/Neu1<tm1Adz> | either: (involves: 129S1/Sv * C57BL/6) or (involves: 129S1/Sv * NMRI) | MGI:3719098 |  | abnormal Kupffer cell morphology | hematopoietic system | J:76937 |
| NEU1 | Neu1<tm1Adz>/Neu1<tm1Adz> | either: (involves: 129S1/Sv * C57BL/6) or (involves: 129S1/Sv * NMRI) | MGI:3719098 |  | abnormal Kupffer cell morphology | immune system | J:76937 |
| NEU1 | Neu1<tm1Adz>/Neu1<tm1Adz> | either: (involves: 129S1/Sv * C57BL/6) or (involves: 129S1/Sv * NMRI) | MGI:3719098 |  | abnormal Kupffer cell morphology | liver/biliary system | J:76937 |
| NEU1 | Neu1<tm1Adz>/Neu1<tm1Adz> | either: (involves: 129S1/Sv * C57BL/6) or (involves: 129S1/Sv * NMRI) | MGI:3719098 |  | abnormal lysosome morphology | cellular | J:76937 |
| NEU1 | Neu1<tm1Adz>/Neu1<tm1Adz> | either: (involves: 129S1/Sv * C57BL/6) or (involves: 129S1/Sv * NMRI) | MGI:3719098 |  | abnormal microglial cell morphology | hematopoietic system | J:76937 |
| NEU1 | Neu1<tm1Adz>/Neu1<tm1Adz> | either: (involves: 129S1/Sv * C57BL/6) or (involves: 129S1/Sv * NMRI) | MGI:3719098 |  | abnormal microglial cell morphology | immune system | J:76937 |
| NEU1 | Neu1<tm1Adz>/Neu1<tm1Adz> | either: (involves: 129S1/Sv * C57BL/6) or (involves: 129S1/Sv * NMRI) | MGI:3719098 |  | abnormal microglial cell morphology | nervous system | J:76937 |
| NEU1 | Neu1<tm1Adz>/Neu1<tm1Adz> | either: (involves: 129S1/Sv * C57BL/6) or (involves: 129S1/Sv * NMRI) | MGI:3719098 |  | abnormal neuron morphology | nervous system | J:76937 |
| NEU1 | Neu1<tm1Adz>/Neu1<tm1Adz> | either: (involves: 129S1/Sv * C57BL/6) or (involves: 129S1/Sv * NMRI) | MGI:3719098 |  | abnormal urine homeostasis | homeostasis/metabolism | J:76937 |
| NEU1 | Neu1<tm1Adz>/Neu1<tm1Adz> | either: (involves: 129S1/Sv * C57BL/6) or (involves: 129S1/Sv * NMRI) | MGI:3719098 |  | abnormal urine homeostasis | renal/urinary system | J:76937 |
| NEU1 | Neu1<tm1Adz>/Neu1<tm1Adz> | either: (involves: 129S1/Sv * C57BL/6) or (involves: 129S1/Sv * NMRI) | MGI:3719098 |  | accumulation of giant lysosomes in kidney/renal tubule cells | cellular | J:76937 |
| NEU1 | Neu1<tm1Adz>/Neu1<tm1Adz> | either: (involves: 129S1/Sv * C57BL/6) or (involves: 129S1/Sv * NMRI) | MGI:3719098 |  | accumulation of giant lysosomes in kidney/renal tubule cells | renal/urinary system | J:76937 |
| NEU1 | Neu1<tm1Adz>/Neu1<tm1Adz> | either: (involves: 129S1/Sv * C57BL/6) or (involves: 129S1/Sv * NMRI) | MGI:3719098 |  | decreased body weight | growth/size/body region | J:76937 |
| NEU1 | Neu1<tm1Adz>/Neu1<tm1Adz> | either: (involves: 129S1/Sv * C57BL/6) or (involves: 129S1/Sv * NMRI) | MGI:3719098 |  | decreased circulating total protein level | homeostasis/metabolism | J:76937 |
| NEU1 | Neu1<tm1Adz>/Neu1<tm1Adz> | either: (involves: 129S1/Sv * C57BL/6) or (involves: 129S1/Sv * NMRI) | MGI:3719098 |  | distended urinary bladder | renal/urinary system | J:76937 |
| NEU1 | Neu1<tm1Adz>/Neu1<tm1Adz> | either: (involves: 129S1/Sv * C57BL/6) or (involves: 129S1/Sv * NMRI) | MGI:3719098 |  | edema | homeostasis/metabolism | J:76937 |
| NEU1 | Neu1<tm1Adz>/Neu1<tm1Adz> | either: (involves: 129S1/Sv * C57BL/6) or (involves: 129S1/Sv * NMRI) | MGI:3719098 |  | enlarged spleen | growth/size/body region | J:76937 |
| NEU1 | Neu1<tm1Adz>/Neu1<tm1Adz> | either: (involves: 129S1/Sv * C57BL/6) or (involves: 129S1/Sv * NMRI) | MGI:3719098 |  | enlarged spleen | hematopoietic system | J:76937 |
| NEU1 | Neu1<tm1Adz>/Neu1<tm1Adz> | either: (involves: 129S1/Sv * C57BL/6) or (involves: 129S1/Sv * NMRI) | MGI:3719098 |  | enlarged spleen | immune system | J:76937 |
| NEU1 | Neu1<tm1Adz>/Neu1<tm1Adz> | either: (involves: 129S1/Sv * C57BL/6) or (involves: 129S1/Sv * NMRI) | MGI:3719098 |  | extramedullary hematopoiesis | hematopoietic system | J:76937 |
| NEU1 | Neu1<tm1Adz>/Neu1<tm1Adz> | either: (involves: 129S1/Sv * C57BL/6) or (involves: 129S1/Sv * NMRI) | MGI:3719098 |  | extremity edema | homeostasis/metabolism | J:76937 |
| NEU1 | Neu1<tm1Adz>/Neu1<tm1Adz> | either: (involves: 129S1/Sv * C57BL/6) or (involves: 129S1/Sv * NMRI) | MGI:3719098 |  | eyelid edema | craniofacial | J:76937 |
| NEU1 | Neu1<tm1Adz>/Neu1<tm1Adz> | either: (involves: 129S1/Sv * C57BL/6) or (involves: 129S1/Sv * NMRI) | MGI:3719098 |  | eyelid edema | growth/size/body region | J:76937 |
| NEU1 | Neu1<tm1Adz>/Neu1<tm1Adz> | either: (involves: 129S1/Sv * C57BL/6) or (involves: 129S1/Sv * NMRI) | MGI:3719098 |  | eyelid edema | homeostasis/metabolism | J:76937 |
| NEU1 | Neu1<tm1Adz>/Neu1<tm1Adz> | either: (involves: 129S1/Sv * C57BL/6) or (involves: 129S1/Sv * NMRI) | MGI:3719098 |  | eyelid edema | vision/eye | J:76937 |
| NEU1 | Neu1<tm1Adz>/Neu1<tm1Adz> | either: (involves: 129S1/Sv * C57BL/6) or (involves: 129S1/Sv * NMRI) | MGI:3719098 |  | hydronephrosis | renal/urinary system | J:76937 |
| NEU1 | Neu1<tm1Adz>/Neu1<tm1Adz> | either: (involves: 129S1/Sv * C57BL/6) or (involves: 129S1/Sv * NMRI) | MGI:3719098 |  | hyperkeratosis | integument | J:76937 |
| NEU1 | Neu1<tm1Adz>/Neu1<tm1Adz> | either: (involves: 129S1/Sv * C57BL/6) or (involves: 129S1/Sv * NMRI) | MGI:3719098 |  | ischuria | renal/urinary system | J:76937 |
| NEU1 | Neu1<tm1Adz>/Neu1<tm1Adz> | either: (involves: 129S1/Sv * C57BL/6) or (involves: 129S1/Sv * NMRI) | MGI:3719098 |  | kyphosis | skeleton | J:76937 |
| NEU1 | Neu1<tm1Adz>/Neu1<tm1Adz> | either: (involves: 129S1/Sv * C57BL/6) or (involves: 129S1/Sv * NMRI) | MGI:3719098 |  | lordosis | skeleton | J:76937 |
| NEU1 | Neu1<tm1Adz>/Neu1<tm1Adz> | either: (involves: 129S1/Sv * C57BL/6) or (involves: 129S1/Sv * NMRI) | MGI:3719098 |  | myoclonus | behavior/neurological | J:76937 |
| NEU1 | Neu1<tm1Adz>/Neu1<tm1Adz> | either: (involves: 129S1/Sv * C57BL/6) or (involves: 129S1/Sv * NMRI) | MGI:3719098 |  | myoclonus | muscle | J:76937 |
| NEU1 | Neu1<tm1Adz>/Neu1<tm1Adz> | either: (involves: 129S1/Sv * C57BL/6) or (involves: 129S1/Sv * NMRI) | MGI:3719098 |  | myoclonus | nervous system | J:76937 |
| NEU1 | Neu1<tm1Adz>/Neu1<tm1Adz> | either: (involves: 129S1/Sv * C57BL/6) or (involves: 129S1/Sv * NMRI) | MGI:3719098 |  | premature death | mortality/aging | J:76937 |
| NEU1 | Neu1<tm1Adz>/Neu1<tm1Adz> | either: (involves: 129S1/Sv * C57BL/6) or (involves: 129S1/Sv * NMRI) | MGI:3719098 |  | respiratory distress | respiratory system | J:76937 |
| NEU1 | Neu1<tm1Adz>/Neu1<tm1Adz> | either: (involves: 129S1/Sv * C57BL/6) or (involves: 129S1/Sv * NMRI) | MGI:3719098 |  | skin edema | homeostasis/metabolism | J:76937 |
| NEU1 | Neu1<tm1Adz>/Neu1<tm1Adz> | either: (involves: 129S1/Sv * C57BL/6) or (involves: 129S1/Sv * NMRI) | MGI:3719098 |  | skin edema | integument | J:76937 |
| NEU1 | Neu1<tm1Adz>/Neu1<tm1Adz> | either: (involves: 129S1/Sv * C57BL/6) or (involves: 129S1/Sv * NMRI) | MGI:3719098 |  | spleen hyperplasia | growth/size/body region | J:76937 |
| NEU1 | Neu1<tm1Adz>/Neu1<tm1Adz> | either: (involves: 129S1/Sv * C57BL/6) or (involves: 129S1/Sv * NMRI) | MGI:3719098 |  | spleen hyperplasia | hematopoietic system | J:76937 |
| NEU1 | Neu1<tm1Adz>/Neu1<tm1Adz> | either: (involves: 129S1/Sv * C57BL/6) or (involves: 129S1/Sv * NMRI) | MGI:3719098 |  | spleen hyperplasia | immune system | J:76937 |
| NEU1 | Neu1<tm1Adz>/Neu1<tm1Adz> | either: (involves: 129S1/Sv * C57BL/6) or (involves: 129S1/Sv * NMRI) | MGI:3719098 |  | tremors | behavior/neurological | J:76937 |
| HSPA1A | Hspa1a<tm1.1Msk>/Hspa1a<tm1.1Msk> | involves: 129S2/SvPas * 129X1/SvJ * C57BL/6 | MGI:3719803 |  | abnormal cell physiology | cellular | J:72943 |
| HSPA1A | Hspa1a<tm1Msk>/Hspa1a<+> | involves: 129S2/SvPas * 129X1/SvJ * C57BL/6 | MGI:3719791 |  | abnormal cell physiology | cellular | J:72943 |
| HSPA1A | Hspa1a<tm1Msk>/Hspa1a<tm1Msk> | involves: 129S2/SvPas * 129X1/SvJ * C57BL/6 | MGI:3719790 |  | abnormal cell physiology | cellular | J:72943 |
| HSPA1A | Hspa1a<tm1Msk>/Hspa1a<tm1Msk> | involves: 129S2/SvPas * 129X1/SvJ * C57BL/6 | MGI:3719790 |  | increased sensitivity to induced cell death | cellular | J:72943 |
| TNXB | Tnxb<tm1b(EUCOMM)Hmgu>/Tnxb<tm1b(EUCOMM)Hmgu> | C57BL/6N-Tnxb<tm1b(EUCOMM)Hmgu>/H | MGI:5797881 |  | decreased blood urea nitrogen level | homeostasis/metabolism | J:211773 |
| TNXB | Tnxb<tm1b(EUCOMM)Hmgu>/Tnxb<tm1b(EUCOMM)Hmgu> | C57BL/6N-Tnxb<tm1b(EUCOMM)Hmgu>/H | MGI:5797881 |  | decreased bone mineral content | skeleton | J:211773 |
| TNXB | Tnxb<tm1b(EUCOMM)Hmgu>/Tnxb<tm1b(EUCOMM)Hmgu> | C57BL/6N-Tnxb<tm1b(EUCOMM)Hmgu>/H | MGI:5797881 |  | decreased circulating alkaline phosphatase level | homeostasis/metabolism | J:211773 |
| TNXB | Tnxb<tm1b(EUCOMM)Hmgu>/Tnxb<tm1b(EUCOMM)Hmgu> | C57BL/6N-Tnxb<tm1b(EUCOMM)Hmgu>/H | MGI:5797881 |  | enlarged heart | cardiovascular system | J:211773 |
| TNXB | Tnxb<tm1b(EUCOMM)Hmgu>/Tnxb<tm1b(EUCOMM)Hmgu> | C57BL/6N-Tnxb<tm1b(EUCOMM)Hmgu>/H | MGI:5797881 |  | enlarged heart | growth/size/body region | J:211773 |
| TNXB | Tnxb<tm1b(EUCOMM)Hmgu>/Tnxb<tm1b(EUCOMM)Hmgu> | C57BL/6N-Tnxb<tm1b(EUCOMM)Hmgu>/H | MGI:5797881 |  | enlarged spleen | growth/size/body region | J:211773 |
| TNXB | Tnxb<tm1b(EUCOMM)Hmgu>/Tnxb<tm1b(EUCOMM)Hmgu> | C57BL/6N-Tnxb<tm1b(EUCOMM)Hmgu>/H | MGI:5797881 |  | enlarged spleen | hematopoietic system | J:211773 |
| TNXB | Tnxb<tm1b(EUCOMM)Hmgu>/Tnxb<tm1b(EUCOMM)Hmgu> | C57BL/6N-Tnxb<tm1b(EUCOMM)Hmgu>/H | MGI:5797881 |  | enlarged spleen | immune system | J:211773 |
| TNXB | Tnxb<tm1Jbrs>/Tnxb<tm1Jbrs> | involves: 129X1/SvJ * C57BL/6 * FVB | MGI:2655647 |  | abnormal skin tensile strength | integument | J:75812 |
| TNXB | Tnxb<tm1Kmat>/Tnxb<tm1Kmat> | involves: C57BL/6 * CBA | MGI:3053869 |  | abnormal cutaneous collagen fibril morphology | integument | J:92472 |
| TNXB | Tnxb<tm1Kmat>/Tnxb<tm1Kmat> | involves: C57BL/6 * CBA | MGI:3053869 |  | abnormal tumor susceptibility | neoplasm | J:92581 |
| TNXB | Tnxb<tm1Kmat>/Tnxb<tm1Kmat> | involves: C57BL/6 * CBA | MGI:3053869 |  | increased susceptibility to induced morbidity/mortality | mortality/aging | J:92581 |
| ABO | Abo<tm1.1(KOMP)Vlcg>/Abo<tm1.1(KOMP)Vlcg> | C57BL/6N-Abo<tm1.1(KOMP)Vlcg>/Ucd | MGI:6492683 |  | abnormal skin morphology | integument | J:211773 |
| ABO | Abo<tm1.1(KOMP)Vlcg>/Abo<tm1.1(KOMP)Vlcg> | C57BL/6N-Abo<tm1.1(KOMP)Vlcg>/Ucd | MGI:6492683 |  | abnormal testis morphology | endocrine/exocrine gland | J:211773 |
| ABO | Abo<tm1.1(KOMP)Vlcg>/Abo<tm1.1(KOMP)Vlcg> | C57BL/6N-Abo<tm1.1(KOMP)Vlcg>/Ucd | MGI:6492683 |  | abnormal testis morphology | reproductive system | J:211773 |
| ABO | Abo<tm1.1(KOMP)Vlcg>/Abo<tm1.1(KOMP)Vlcg> | C57BL/6N-Abo<tm1.1(KOMP)Vlcg>/Ucd | MGI:6492683 |  | small testis | endocrine/exocrine gland | J:211773 |
| ABO | Abo<tm1.1(KOMP)Vlcg>/Abo<tm1.1(KOMP)Vlcg> | C57BL/6N-Abo<tm1.1(KOMP)Vlcg>/Ucd | MGI:6492683 |  | small testis | reproductive system | J:211773 |
